# Development of Liquid Organic Hydrogen Carriers for Hydrogen Storage and Transport

**DOI:** 10.3390/ijms25021359

**Published:** 2024-01-22

**Authors:** Thi-Hoa Le, Ngo Tran, Hyun-Jong Lee

**Affiliations:** 1 Department of Chemical and Biological Engineering, Gachon University, 1342 Seongnamdaero, Sujeong-gu, Seongnam-si 13120, Gyeonggi-do, Republic of Korea; lehoa290792@gachon.ac.kr; 2 Institute of Research and Development, Duy Tan University, Da Nang 550000, Vietnam; 3 Faculty of Natural Sciences, Duy Tan University, Da Nang 550000, Vietnam

**Keywords:** liquid organic hydrogen carriers, catalysts, dehydrogenation, hydrogenation, reactors

## Abstract

The storage and transfer of energy require a safe technology to mitigate the global environmental issues resulting from the massive application of fossil fuels. Fuel cells have used hydrogen as a clean and efficient energy source. Nevertheless, the storage and transport of hydrogen have presented longstanding problems. Recently, liquid organic hydrogen carriers (LOHCs) have emerged as a solution to these issues. The hydrogen storage technique in LOHCs is more attractive than those of conventional energy storage systems like liquefaction, compression at high pressure, and methods of adsorption and absorption. The release and acceptance of hydrogen should be reversible by LOHC molecules following favourable reaction kinetics. LOHCs comprise liquid and semi-liquid organic compounds that are hydrogenated to store hydrogen. These hydrogenated molecules are stored and transported and finally dehydrogenated to release the required hydrogen for supplying energy. Hydrogenation and dehydrogenation are conducted catalytically for multiple cycles. This review elaborates on the characteristics of different LOHC molecules, based on their efficacy as energy generators. Additionally, different catalysts used for both hydrogenation and dehydrogenation are discussed.

## 1. Introduction

The depletion of fossil fuels has forced humans to search for alternative energy sources like renewable energy systems derived from the wind, sun, oceans, etc. [1]. However, the energy obtained from renewable sources is not consistent since these sources fluctuate under seasonal and meteorological conditions. These situations may lead to a shortage of power in unfavourable weather conditions and produce additional power in favourable weather, which hinders the production of a stable energy system. The electrolysis of water is another technique of saving electricity through the generation of hydrogen [2]. The gravimetric energy density of hydrogen storage is 120 MJ Kg^−1^, which can be converted into electrical energy without producing any toxic emissions [3]. Thus, hydrogen has been depicted as a clean energy resource to replace fossil fuels in the coming decades.

The prospect of the application of hydrogen as a next-generation fuel has initiated enormous research activities in this area. However, the highly explosive and flammable property of hydrogen stands as an obstacle in the development of a hydrogen economy. Another issue is the development of an appropriate storage system, since the volumetric density of hydrogen is very low. Consequently, the storage of hydrogen has also become an area of research interest. Hydrogen is stored under high pressure, which requires heavy infrastructure. A huge amount of energy and investment in compression technology is required to store hydrogen in its liquid form in cryogenic storage [4]. Materials with high surface areas, such as carbon nanotubes and metal–organic frameworks, have also been used for storing hydrogen, but the associated cooling process is tedious [5]. Although the solid metal hydrides have been promising candidates, they exhibit low gravimetric loading and also degrade during the cycling process [6].

Liquid organic hydrogen carriers (LOHCs) have sparked immense interest in both industrial as well as academic fields, based on their potential for hydrogen storage and transportation [7], safety, capability to undergo reversible dehydrogenation and hydrogenation [8], cheap [9] and facile thermal management [10], and strong compatibility with the present infrastructure of gasoline [11]. The H lean form specifically signifies the LOHC and constitutes mainly polycyclic organic aromatic compounds. The release and storage of hydrogen gas within organic compounds are very slow processes, and hence, are accelerated in the presence of catalysts. The transportation of hydrogenated LOHCs is feasible at atmospheric pressure, utilizing the existing transportation facilities. The volumetric density is 50 g L^−1^, while the gravimetric density is over 5 wt% for hydrogen stored using the LOHC storage techniques. The dehydrogenation of the LOHCs releases hydrogen which is free of unrequired gases like CO_2_, CO, and their by-products.

**Figure 1:**
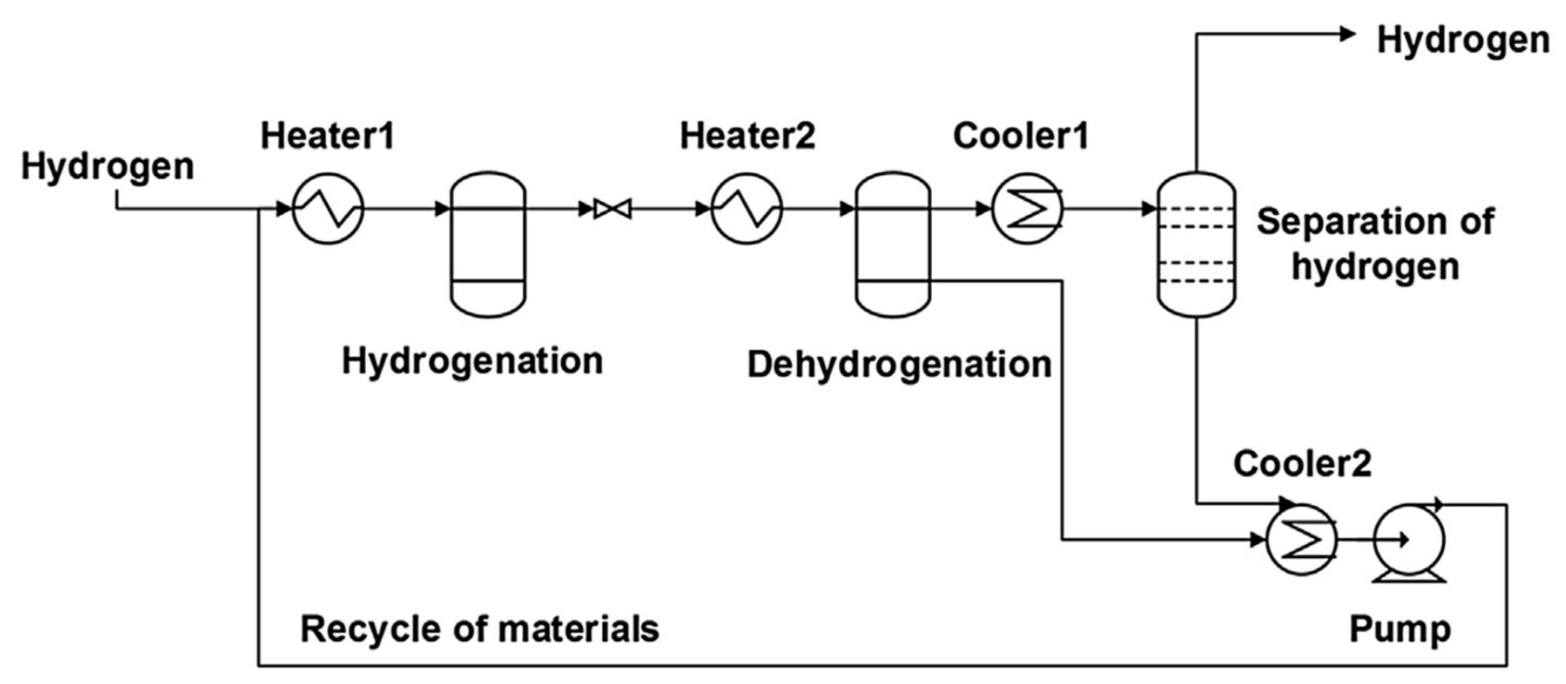
Schematic representation of the LOHC process. Reproduced with permission from [15].

An LOHC technology was reported by Sultan and Shaw in 1975 that applied liquid aromatic compounds (benzene (gravimetric hydrogen content of 7.19 wt%); naphthalene (gravimetric hydrogen content of 7.23 wt%); toluene (gravimetric hydrogen content of 6.18 wt%)) as carriers of hydrogen [12]. The dehydrogenation temperature of these compounds is more than 300 °C, which is above the operating temperature of a fuel cell and is also associated with numerous side reactions and sluggish reaction rates. The next development in this field was the discovery of heteroaromatic compounds which possessed a dehydrogenation temperature below 200 °C [13]. The temperature was decreased by the introduction of heteroatoms like nitrogen and sulphur into the aromatic ring. Carbazole-based compounds, particularly N-ethylcarbazole (NEC), have been accepted as promising candidates for LOHCs based on their inherent properties like their low temperature for dehydrogenation and high amount of hydrogen [14]. The hydrogen storage capacity of NEC is 5.8 wt% and its hydrogenated form is dodecahydro-N-ethylcarbazole (H12-NEC). The process of hydrogen storage is shown in Figure 1.

Ru catalysts have demonstrated effective activity in the hydrogenation reaction of LOHCs under mild conditions [16]. Metal catalysts based on Pd, Pt, and Rh along with Al_2_O_3_ as supports are active in the dehydrogenation of LOHCs [17]. A complete dehydrogenation was obtained with Pd and Pt as catalysts. Dodecahydro-N-ethylcarbazole can be dehydrogenated below 200 °C to generate hydrogen in its pure form [18]. Although NEC has been established as an effective hydrogen carrier, there still exist certain obstacles to its application on a large scale, such as enhancing the rate of dehydrogenation and decreasing the reaction temperature, which can be solved through further research.

After an elaborate search, we found that although there are few reviews on LOHC regarding its progress [19,20] and different catalysts for the hydrogenation and dehydrogenation of N-ethylcarbazole [21], we decided to summarise the recently reported molecules and the characteristics required for their hydrogen, dehydrogenation, and storage. This review focuses on the development of the LOHCs along with the progress made with different catalysts in accelerating the process of generating hydrogen.

### 1.1. Physical and Chemical Method of Hydrogen Storage

There is no linear connection of the volumetric density of hydrogen and pressure [22]. The energy needed for hydrogen gas compression, coupled with its low density, poses challenges for efficient hydrogen transport [23]. The liquefaction of hydrogen can enhance the density. The density of liquid hydrogen is 70.8 kg/m^3^ at 21 K [24]. There is a loss of hydrogen due to the transfer of the heat through containers as the critical temperature of hydrogen is 33 K. Additionally, the cooling system adds extra weight and also utilizes energy. Thus, the system should be equipped with strong thermal insulators to decrease the loss of hydrogen through vaporization [25]. The theoretical density of hydrogen to be stored should be high for efficient storage capacity. The liquefaction of hydrogen gas is not feasible as it uses one-third of the total amount of energy stored in hydrogen [26]. Another route, for the physical storage of hydrogen, is by adsorption into porous materials with high surface areas like activated carbons, a covalent organic framework, or a metal–organic framework [27]. However, the capacity of hydrogen storage is less with these materials.

To overcome the limitation of the physical storage of hydrogen, the chemical storage of hydrogen has evolved to make the transportation of stored hydrogen economically feasible on a large scale. The densities of hydrogen in chemical storage are enhanced to 170 kg H_2_/m^3^, as hydrogen forms covalent bonds which are shorter [26]. The release and storage of hydrogen from the organic liquids are carried out through the dehydrogenation and hydrogenation process. The liquefaction and compression processes are eliminated, since hydrogen molecules are stored by the formation of covalent bonds. Hydrogenated LOHC is dehydrogenated catalytically on-board, on the vehicles for releasing the stored hydrogen. The used (dehydrogenated) fuel is preserved in a storage tank which is later re-generated off-board by hydrogenation [28]. The main criteria of LOHC are that it must possess a low melting point and a high boiling point, such that it retains its liquid phase at a low temperature. Additionally, LOHC compounds should not decompose and should undergo dehydrogenation and hydrogenation selectively [29,30]. However, there are challenges to meet the requirements of the storage capacity, as the temperature required for the dehydrogenation reaction is very high and inconvenient during transportation. This step has been improved by the introduction of a heteroatom into the aromatic ring [13]. The presence of nitrogen in five- or six-membered heterocycles reduces the heat of hydrogenation, enhancing this crucial step.

### 1.2. Thermodynamics of Hydrogen Storage

The probability of hydrogenation and dehydrogenation of an organic molecule to be used as a hydrogen carrier is dictated by the thermodynamics of the reaction. Notably, the hydrogenation process is thermodynamically favourable, while the process of dehydrogenation is difficult. This is because both the processes possess the same free energy of reaction with different algebraic signs. The free energy of the reaction is given by the following Equation (1).

(1) ∆G=∆H+T∆S

where

∆G = free energy of the reaction;
∆H = enthalpy of the reaction;
∆S = entropy of the reaction;
T = temperature.

Although the hydrogenation reaction is not favoured by entropy, it is driven by the equilibrium of the reaction. The free energy of the reaction [36 KJ (mol H_2_)^−1^] exceeds that of the enthalpy of the reaction. The hydrogenation of unsaturated compounds is an exothermic reaction and hence is thermodynamically propelled at room temperature, although not feasible due to entropy. Elevating the temperature shifts the equilibrium toward dehydrogenation. Thus, the organic compounds having an enthalpy of reaction lower or equal to −40 KJ (mol H_2_)^−1^ should be selected for LOHC, as this will make the process of dehydrogenation feasible. A higher enthalpy of reaction requires the temperature to be lower than 20 °C to pass the thermodynamic barrier. Thus, the kinetics of the reaction will be sluggish. Conversely, the temperature required for the dehydrogenation reaction will be very high, if the enthalpy of the reaction is lower than the desired value of −40 KJ (mol H_2_)^−1^. The thermodynamic properties of some organic compounds as potential LOHCs are shown in Table 1.

Organic compounds with isolated double bonds can be easily hydrogenated, while their dehydrogenation is very tough. The hydrogenation of ethene is thermodynamically feasible to form ethane, while its dehydrogenation is not viable. Compounds containing conjugated double bonds are a better choice as LOHC, but with a feeble force for initiating the dehydrogenation reaction. The dehydrogenation process is viable only to form aromatic compounds since they are highly stable. The dehydrogenation to form double bonds outside the aromatic ring, although conjugated, requires high energy. The thermodynamic properties of a ring are altered by the introduction of a heteroatom. Nitrogen positively enhances dehydrogenation, while sulphur and oxygen have negative impacts. The presence of boron along with nitrogen propels the dehydrogenation reaction further. The temperature can be further decreased by diminishing the partial pressure of the system or by the addition of inert gas. Nevertheless, the quality of hydrogen is affected by these methods.

### 1.3. Techniques of H Storage

A comparative study on the high-pressure hydrogen storage techniques and LOHC was conducted which revealed that the efficiency of the LOHC system was 69.17% (without recycling) or 88.74% (by recycling) depending on the heat produced during the hydrogenation process. However, the efficiency of energy for methods using high-pressure storage was higher as compared to that of the LOHC [31].

Hydrogen molecules are adsorbed through physical adsorption on materials with large surface areas like active carbon, zeolites, and carbon nanotubes while hydrogen adsorbs to metal surfaces through chemisorption. The peculiarity of organic liquid carriers is that they maintain a liquid state through all the stages of dehydrogenation and hydrogenation [32]. The storage efficacy is decreased when the hydrogen is stored for a short time [33]. The reversible process of hydrogenation and dehydrogenation, in the presence of catalysts, is used for the addition and removal of hydrogen from the LOHC. The heterocyclic aromatic compounds were considered as LOHCs, as the presence of a hetero atom reduced the enthalpy required for dehydrogenation due to diminishing the aromaticity [34]. The optimal enthalpies of the exothermic hydrogenation of LOHCs are within a range of 9.56–16.73 kcal mol^−1^ [35]. The hydrogenation is carried out at a temperature range of 373–573 K and a pressure of 1–5 MPa in the presence of catalysts (Ru or Ni on oxide support), on trickle bed units or slurry phase tanks [36]. The dehydrogenation of LOHCs is an endothermic process that takes place at a temperature of 423–673 K and a pressure of 1–0.5 MPa, in the presence of catalysts (Pt, Pd) in tubular reactors or tanks equipped with a slurry phase [37]. The hydrogen lean LOHC is extracted from the dehydrogenation reactor through condensation and is stored for its next cycle. A few LOHCs are listed in Table 2.

The requisite heat of dehydrogenation of pure hydrocarbon can be obtained from the heat liberated during the hydrogenation of compounds in a stationary energy storage system. This process is feasible only when catalytic hydrogenation and dehydrogenation can take place in the same hot reactor. The alicyclic dehydrogenation should be carried out at a lower temperature than that of the aromatic hydrogenation for maintaining the heat of hydrogenation. This heat is stored and reused further in the process of dehydrogenation. The conventional storage system for LOHC is equipped with individual reactors for the hydrogenation and dehydrogenation process. The dehydrogenation reactors work at a low pressure and high temperature, while the hydrogenation reactor works at a high pressure and low temperature for maintaining the thermodynamic forces [38]. This process leads to the wastage of a large amount of heat while heating the dehydrogenation reactor from room temperature to the temperature required for the dehydrogenation process and vice versa. Thus, the hot hydrogenation reactor is a good option for carrying out the dehydrogenation process, which would save the additional wastage of heat. According to thermodynamic principles, exothermic hydrogenation should occur at low temperatures, while endothermic dehydrogenation should occur at a higher temperature. The reaction equilibrium of an LOHC system is highly dependent on pressure, boosting hydrogenation and dehydrogenation. Pressure swing reactors aid in the release of hydrogen even when the reactor operates at the same temperature for both the dehydrogenation and hydrogenation processes.

The storage of hydrogen in dibenzyl toluene (DBT) was studied using a hot pressure swing reactor [39]. Both the process of hydrogenation and dehydrogenation were carried out in the presence of Pt on alumina as a catalyst (0.015 mol%) at 290 °C. The temperature for hydrogenation was enhanced above the dehydrogenation temperature for transporting the heat of hydrogenation to the device for storing heat (latent heat storage system like molten salt) for being used in the dehydrogenation process. The flow of heat from the hydrogenation reactor to the storage system and again to the reactor was feasible with a temperature difference of 10 °C. The hydrogenation was above 0.95, while the release of hydrogen was below 0.3. The Pt catalyst was regenerated during the alternate process. The deposits of carbon on the Pt surface during the dehydrogenation at low pressure were removed during the hydrogenation process. However, the LOHC carrier molecule has the possibility of degrading its chemical and physical properties due to harsh storage conditions.

### 1.4. The Risks Linked with the LOHC Systems

A detailed assessment of the hazards related to LOHC technology was conducted on eco-toxicological parameters as well as chemical parameters like partition coefficients, volatility, solubility, etc. [40]. A mixture of LOHCs in a system consisting of isomers lower the melting point of the mixture. However, the analysis of the toxicological properties of the mixture becomes extremely difficult since these compounds are insoluble in water. However, the mixture of these isomers interacts much less as compared to the production on a large scale and thus, exhibits little effect on the system. Additionally, this LOHC may consist of impurities of the substrates and a mixture of compounds hydrogenated to different degrees. Moreover, the recycling process degrades the compounds to a considerable extent. Again, experimental handling is difficult for compounds possessing elevated partition coefficients for octanol and water with low aqueous solubility, as the concentration cannot be maintained at the desired value. The appropriate concentration of the hydrophobic compounds should be accounted for in the proposed LOHC structures in order to avert chronic toxicity in living organisms. Thus, the LOHC with the least toxicity should be accounted for, heeding the environmental concern. As compared to crude oil, the risk management of LOHCs is nominal, since the background of the molecules is familiar. LOHCs should always be designed considering the eco-toxicological profile including the mutagenic, carcinogenic, and toxic. However, these parameters are challenging to predict by the quantitative structure–activity relationship (QSAR). The hydrogen lean compounds are highly biodegradable as compared to their hydrogenated counterparts. The genotoxic actions of some LOHC (polyaromatic hydrocarbons) involve the intercalation of DNA related to the formation of adducts, as they have the tendency toward electrophilic attack and an inherent planar geometry.

The toxic and mutagenic activity of azaarenes (carbazoles and quinolones) is due to their hydrophobicity [41]. The introduction of specific substituents at certain positions on aromatic rings can alter the mutagenicity of the compounds. The presence of an ethyl group or methyl group on the aromatic ring does not affect the mutagenicity, while the same at the nitrogen atom of the carbazole increases the mutagenicity in the reverse mutation assay of Salmonella bacteria [42]. The bacterial mutagenicity, toxicity, and biodegradable nature were studied for four LOHCs (quinaldine, ethylcarbazole, propylcarbazole, and butylcarabazole system) [43]. The hydrogen-rich form, dehydrogenated form, and partially hydrogenated form were analysed as LOHC systems. Among the three types of aquatic toxicity formulated by the globally harmonised system, the hydrogenated LOHC was found to be toxic for Daphnia magna, while the hydrogen lean forms of the alkyl carbazole exhibited minimal toxicity to aquatic species. These LOHCs degenerated after numerous cycles (approximately 10 cycles) of hydrogenation and dehydrogenation depending on the reaction condition and morphology of the catalysts. The degeneration of LOHCs was influenced by the morphology of the catalysts and reaction conditions. For example, the hydrogen storage capacity of ethylcarbazole in the presence of the Ru/Al_2_O_3_ catalyst was reduced by 2% at 80 bar and 180 °C [43]. These degenerated products are toxic to aquatic life.

### 1.5. Kinetics of Hydrogen Storage

The kinetics of storing hydrogen was studied using NEC-doped fullerene due to the appropriate combination of geometric and electronic structures [44]. The hydrogen storage reaction of NEC-doped fullerene is shown in Equation (2).

(2) $$NEC@C_{60}+6H_{2}\to 12H-NEC@C_{60}$$

The analysis of the intake of hydrogen by fullerene gave a value of ΔG as 9.1 kcal mol^−1^, which makes the reaction thermodynamically unfeasible, although the activation energy is decreased [45]. However, the interaction of hydrogen molecules with p electrons favours the storing of hydrogen by NEC@C_60_. The doping of NEC in fullerene enhanced the rate of the reaction, as the activation energy was reduced. Carbon atoms present in the C-N bonds behave as donors, while the nitrogen atom is the electron acceptor.

### 1.6. Effect of Melting Points of N-Alkyl Carbazoles

**Scheme 1:**
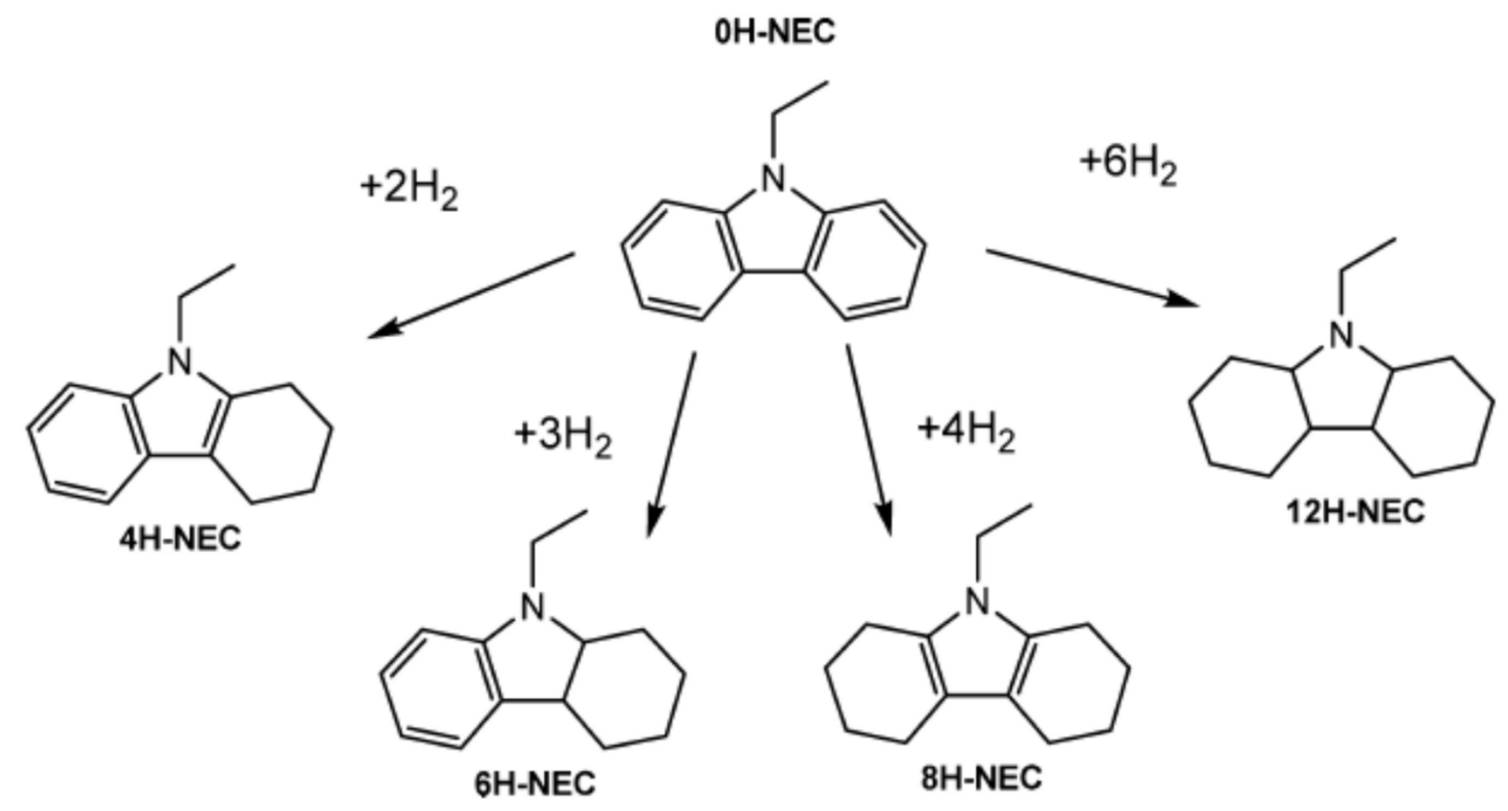
Different forms of hydrogenated NEC. Reproduced with permission from [46].

NEC is the compound having the highest melting point among the different LOHCs. The hydrogenation of NEC leads to the formation of different compounds as shown in Scheme 1. These different mixtures of partially hydrogenated compounds complicate the crystallization nature of the mixture.

The length of the alkyl chain determines the melting point of the N-alkyl carbazoles [47]. A highly branched isopropyl carbazole has a higher melting point than that of n-propyl carbazole. A study on the melting point of different hydrogenated forms of NEC, as well as different alkyl carbazoles, was conducted [48]. It was found that the melting point of 4H-NEC was lower than that of 8H-NEC. Additionally, the melting points of both the compounds were lower than that of 0H-NEC. Since carbazole is the main impurity present in NEC, an evaluation of the melting points of hydrogenated carbazoles revealed that the melting point decreased with an increase in the number of hydrogen atoms added to the carbazole moiety. Again, the enthalpy of fusion of carbazole is more as compared to the NEC and N-methyl carbazole cases. The eutectic nature was found to be ideal for the binary mixtures (NEC-N-methyl carbazole, N-methyl carbazole–carbazole, NEC-N-butylcarbazole, NEC-N-propylcarbazole). Thus, the storage capacity can be effectively maintained by using binary mixtures of N-alkyl carbazoles of which NEC-NPC with a 5.6 weight % of hydrogen storage capacity was found to be optimum. The ternary eutectic points were also explored which established the fact that mixtures of N-alkyl carbazoles are promising alternatives in place of pristine NEC for storing hydrogen.

## 2. LOHC Molecules

Hydrogen eliminated from LOHC is pure which has enhanced the demand for new LOHCs, more susceptible to hydrogenation and dehydrogenation. Bicyclic heteroatomic compounds are highly favoured because of their reduced aromaticity [49]. Additionally, 5-membered rings with a nitrogen atom at the C-1 position is favoured, since the C-H bond is stronger than the N-H bond [25]. Moreover, the rate of dehydrogenation is augmented due to steric hindrance around nitrogen atoms [50]. Although, nitrogen atoms at C-1 and C-3 are highly preferred, the imidazole molecule prevents hydrogenation. The following is a list of molecules as potential LOHCs.

### 2.1. N-Alkylcarbazole

The promising candidates for LOHCs are evaluated using simulation and molecular modelling, instead of practically synthesizing the molecules and analysing their properties. The feasibility of the potential as an LOHC was studied for (N-propyl, N-isopropyl, and N-butyl) carbazole by hydrogenating them completely to (12H-Npropyl, 12H-Nbutyl) carbazole through a computational study [25]. The vapour pressure of N-alkyl carbazoles is substantially decreased by elongating the length of the alkyl chain. However, the enthalpy of vaporization of NEC was lower than that of the aforementioned substrates. The N-alkyl carbazoles possess rigid and flat structures, while their hydrogenated derivatives are flexible. The lower enthalpy of reaction further favours these compounds as potential LOHC molecules.

**Figure 2:**
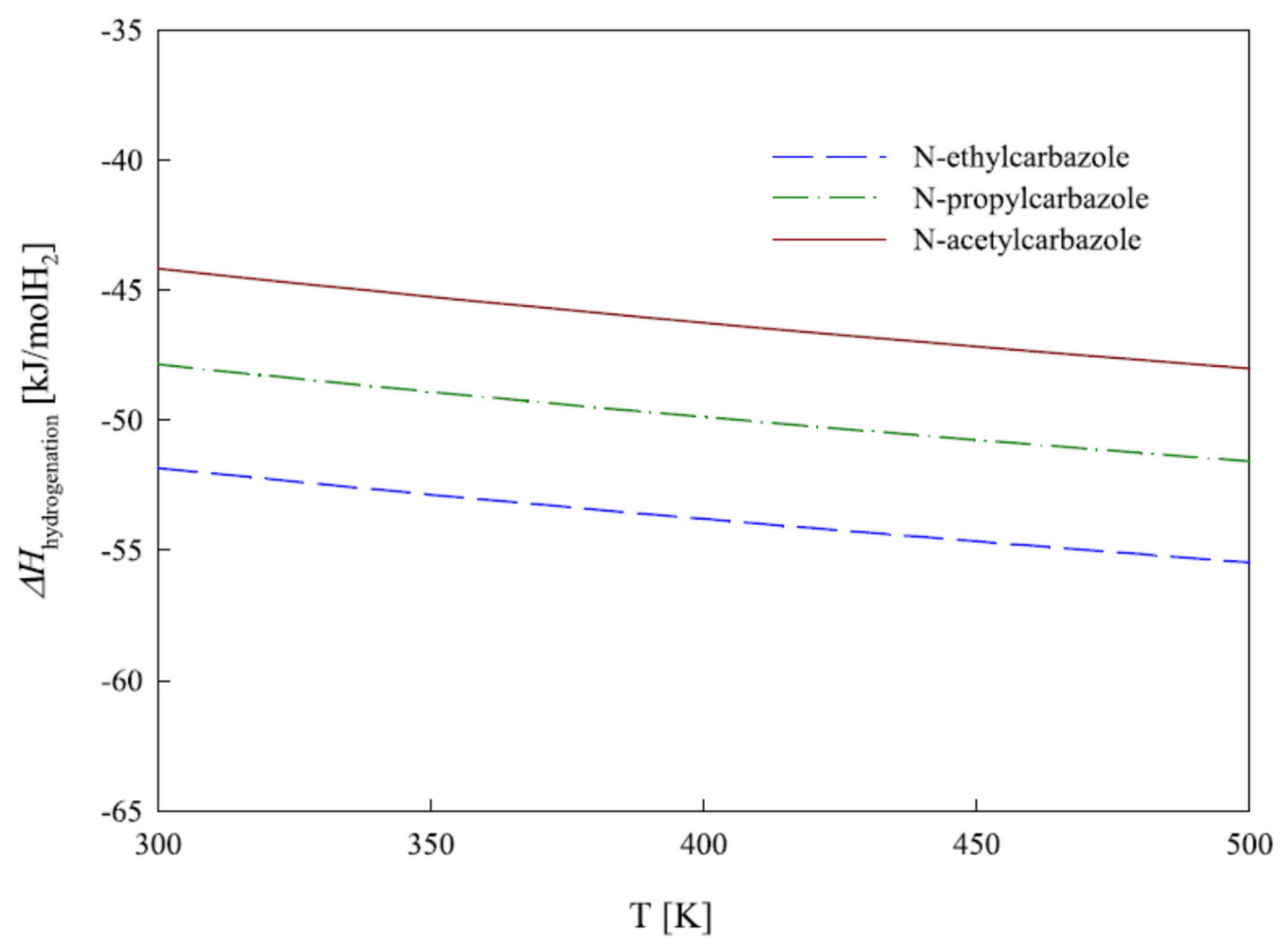
The enthalpy of hydrogenation of three compounds. Reproduced with permission from [15].

In another study, DFT calculation was carried out to study the thermodynamic and physical properties of propylcarbazole, ethylcarbazole, and n-acetylcarbazole, which indicated that the endothermic dehydrogenation reaction required a temperature of 473 K [15]. The requirement of energy in a reaction system is dictated by the cooling and heating process of the reaction system. It was found from the mathematical analysis that the amount of heat that evolved or used during the hydrogenation and dehydrogenation process was lowest for acetyl carbazoles, since the formation of C=O was easier than the dehydrogenation of the ring. Figure 2 illustrates the enthalpy of hydrogenation.

#### 2.1.1. N-Ethyl Carbazole

**Scheme 2:**
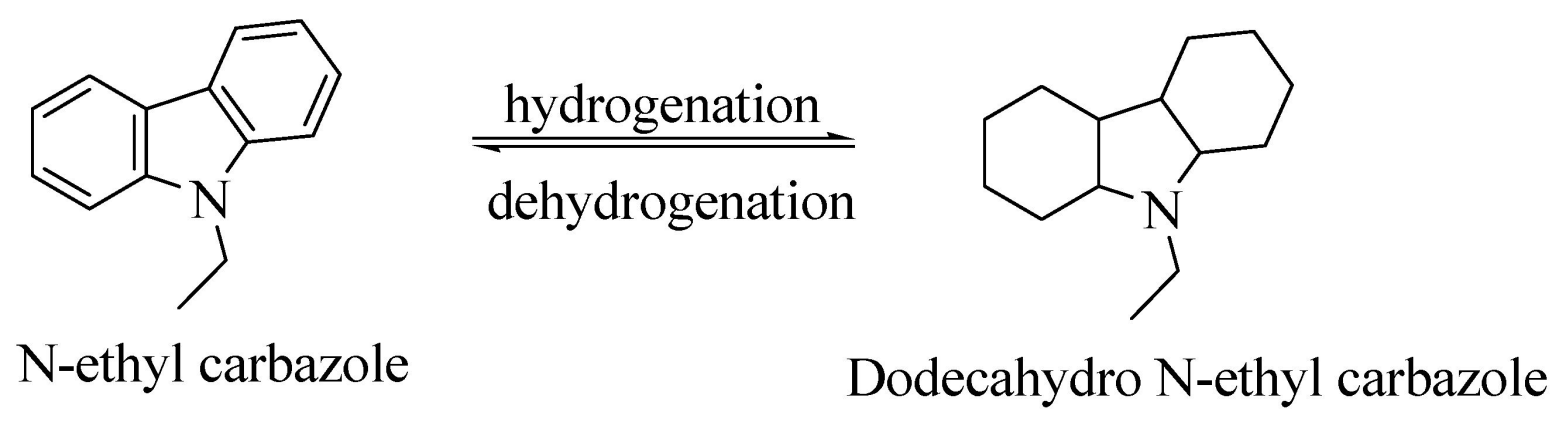
The hydrogenation and dehydrogenation process of NEC. Reproduced with permission from [47].

N-ethyl carbazole (NEC) is an aromatic heterocyclic compound that is used for storing hydrogen with a hydrogen storage capacity of more than 5.8 weight %. The boiling point of completely hydrogenated NEC is around 200 °C [50]. The melting point of carbazole is decreased from 243 °C to 60 °C after the introduction of an ethyl group, making the process of hydrogenation reversible under solvent-free conditions [47]. The process of hydrogenation and dehydrogenation of NEC is shown in Scheme 2.

The mechanism of hydrogenation of NEC was studied computationally using a model of the atomic charges, the energy of localization. and atoms present in the molecule [51,52]. The approach of hydrogen molecules towards NEC was studied with varied orientations, and it was found that hydrogen approached the molecule by a transoid conformation concerning the ethyl group. The activation energy is enhanced due to steric repulsion by the ethyl group at the cis position. Hydrogen remains in the vicinity of the C_11_-C_12_ bond under this condition, but the transition state reveals that the H should attack the C_10_-C_11_ bond, indicating that the H attacks the position, free of steric hindrance. The use of decalin as a solvent hastened the process of hydrogen storage.

**Scheme 3:**
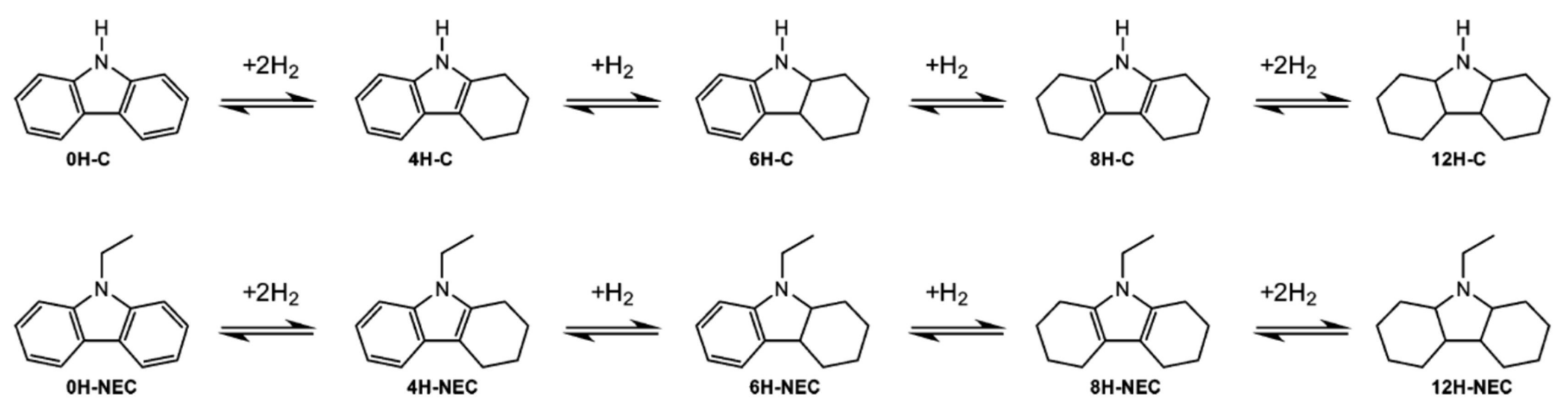
The reaction intermediates of carbazole and NEC. Reproduced with permission from [14].

The major intermediates formed during the process of hydrogenation and dehydrogenation are shown in Scheme 3 [14]. A computational study was conducted to investigate the intermediates formed by carbazole and NEC during the process of catalytic dehydrogenation and hydrogenation. The parameters like the viscosity, refractive index, and surface tension of the intermediate molecules are reduced with the increasing degree of hydrogenation.

**Figure 3:**
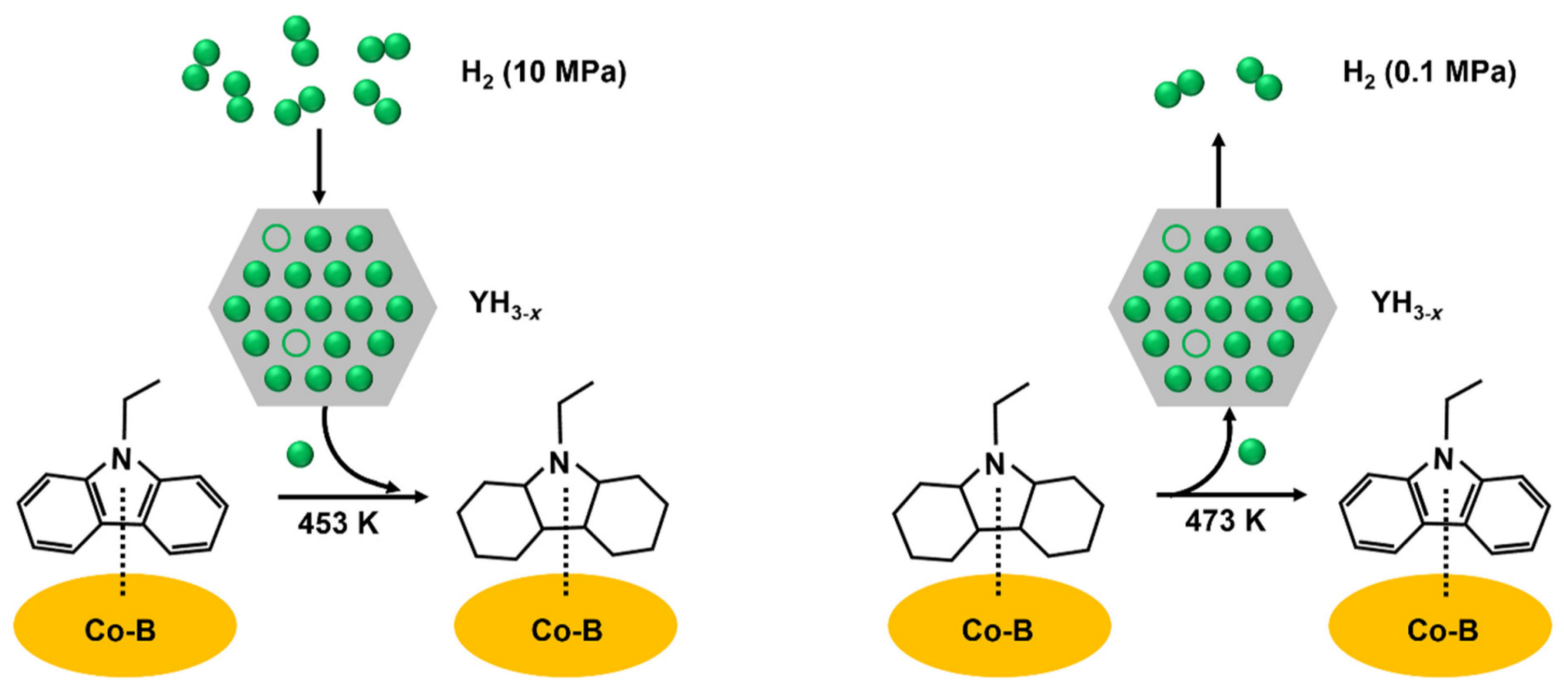
The process of hydrogenation and dehydrogenation. Reproduced with permission from [53].

The reversible storage of hydrogen in NEC/12HNEC in both directions was conducted using cobalt boride blended with yttrium trihydride which was adhered to an alumina support [53]. The LOHC molecules should be appositely activated with a faster rate of hydrogen transfer for the optimal process of hydrogenation and dehydrogenation. The vacant sites for hydrogen bonding and the hydrogen atoms should be near the activated LOHC molecule. The nonstoichiometric YH_3−x_ offers a huge reservoir of hydrogen bonding sites and atomic hydrogen along with rapid kinetics for the adsorption and desorption of hydrogen. However, the synergism of YH_3−x_ is essential with Co-B for both the dehydrogenation and hydrogenation process, where the LOHC molecule was activated by Co-B, while the rate of hydrogen transfer was enhanced by YH_3−x_ as shown in Figure 3. The LOHC molecule was activated by the migration of hydrogen between the interface of YH_3−x_ and Co-B. The two-direction transfer of hydrogen can take place only in the presence of nonstoichiometric YH_3−x_ consisting of octahedral interstices which are not occupied.

The kinetics of dehydrogenation of 12H-NEC was studied using a 5 weight % of Pd catalysts supported on alumina at 101 kpa and a temperature range of 140–170 °C [54]. The ratio of the mass of the dehydrogenation catalyst to that of the reactant was 1:20. Three stable isomers of 12H-NEC were studied which revealed that only one isomer was rapidly converted to its completely dehydrogenated product. The three isomers were dehydrogenated in three steps. The activation energy of all three isomers was different. The second step was difficult, followed by the third step of dehydrogenation which was the most difficult and rate-limiting step.

The catalytic dehydrogenation of 12H-NEC was studied by supporting Pd over different supports which showed that Pd supported on C had the highest activity, while the same supported on SiO_2_ exhibited the lowest activity [48,55]. The high rate of dehydrogenation with Pd/C was due to the large pore volume and specific surface area which augmented the prospect of contact between the catalyst, reactants, and intermediates. Although the smaller size of Pd is crucial for effective catalytic activity [56], Pd on supports with smaller sizes was not found to be effective. This was because, apart from the size of the Pd particle, the structure of the supported surface along with the degree of reduction of Pd plays a significant role in the activity of the catalyst. Thus, the order of catalytic activity was Pd loaded on C, Al_2_O_3_, TiO_2_, and SiO_2_ among the investigated supports. The degree of reduction of Pd was highest for Pd/SiO_2_ because of the existence of available active sites for the reaction. However, the size of Pd particles on SiO_2_ was larger and Pd agglomerated on the surface decreased the catalytic activity.

#### 2.1.2. N-Propyl Carbazole

The hydrogenation of N-propyl carbazole was conducted in the presence of a 5% Ru_2.5_Ni_2.5_/Al_2_O_3_ catalyst. The pore sizes of alumina in the catalyst were decreased as the active sites were occupied by Ni and Ru nanoparticles [57]. Additionally, the catalysts were well dispersed enhancing the efficacy of the catalysts. The interaction between the Ni and Ru is very strong augmenting the ability of the catalyst to absorb hydrogen. The hydrogen bond is dissociated by Ru to form hydrogen atoms which are transferred to the surface of alumina and Ni. Thus, the rate of hydrogenation is increased. An abundance of electrons is maximum on Ni, while Ru is the electron-deficient atom. The distribution of charges on the clusters dictates the interaction capability with hydrogen [58].

### 2.2. 2-Amino Ethanol

The compound 2-aminoethanol was obtained by hydrogenation of cyclic dipeptide, later dehydrogenated in the presence of a Ru pincer catalyst to again furnish the cyclic dipeptide [59]. Thus, hydrogen can be removed and added as per the requirement. This process followed a dehydrogenative coupling process. Both the process of hydrogenation and dehydrogenation take place in the presence of the same catalyst. This process has highlighted the system of acceptor-less formation of peptide bonds to form LOHCs.

### 2.3. Dibenzyl Toluene (DBT)

Benzyl toluene and dibenzyl toluene (DBT) have been investigated as LOHCs [60], which revealed that the density and surface tension of the hydrogenated compounds is lower, while the viscosity is higher than that of the parent compounds due to the planar aromatic rings in the dehydrogenated compounds. Although the hydrogenation of benzyl toluene and dibenzyl toluene is favoured as compared to NEC, the dehydrogenation process of NEC is more favourable.

The performance of dibenzyl toluene was enhanced by the application of waste heat in a cement plant for the dehydrogenation process [61]. The temperature of the exhaust heat in a cement plant ranges between 200 and 600 °C. The thermal efficiency of the system was enhanced by 28.5% by adding a storage system for this thermal energy. Thus, the cost of electricity can be decreased substantially in this system.

**Figure 4:**
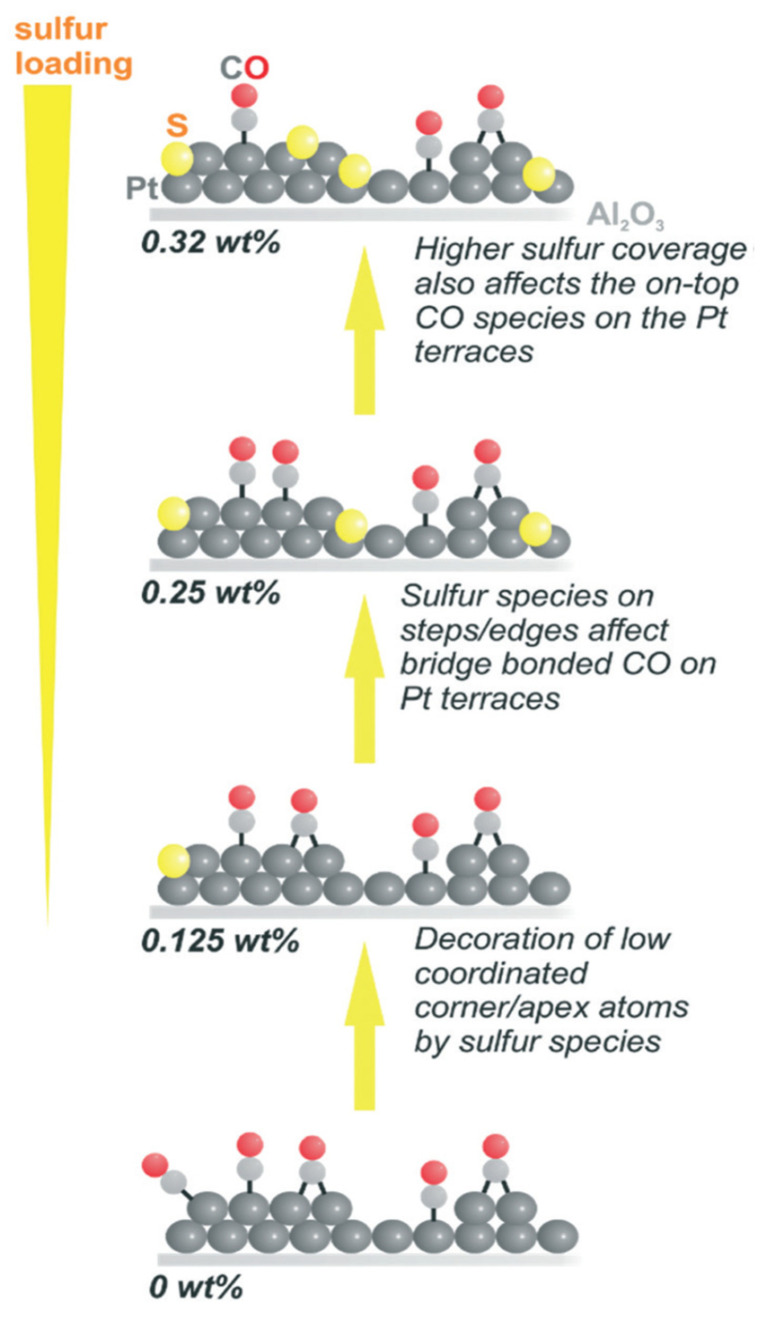
The presence of sulphur on the Pt catalyst. Reproduced with permission from [62].

The dehydrogenation of H18-dibenzyl toluene was carried out by using Pt on an alumina catalyst modified with sulphur (0.25 weight %), which decreased the formation of side products with high boiling points and completed the reaction within 40 min [62]. However, loading a greater amount of sulphur than 0.25% decreases the activity of the catalyst. The sites on the surface of Pt were blocked selectively by the addition of sulphur, as it is adsorbed on the corners and edges initially, followed by adsorption on Pt terraces. Thus, the CO molecules connected to Pt terraces are also affected as shown in Figure 4. The catalytic efficiency was augmented as the defect sites with low coordination were blocked, and the desorption was also promoted as electrons are attracted by sulphur from Pt, thereby decreasing the adsorption rate. The process of desorption is appropriate for the regeneration of the active site of the catalyst for the following cycles. There is a strong tendency of aromatic compounds to be adsorbed strongly, which was considerably decreased by sulphur [63]. Additionally, sites at the Pt terrace are significant.

DBT was hydrogenated using Raney-Ni as the catalyst at a pressure of 0.80 MPa and a temperature of 170 °C [64]. Dehydrogenation was effectively performed at 290 °C in the presence of 1% Pt/C as the catalyst.

### 2.4. Cyclohexane

Bimetallic Ni-Cu was supported on SBA-15 for the dehydrogenation of cyclohexane, where the size of the crystal increased with an increase in the Cu content and pore diameter of SBA-15. The selectivity to form benzene is enhanced with an increase in the amount of Cu in the bimetal, as Ni assembles with a lower coordination number and hinders the formation of methane. The catalytic efficacy is increased due to the formation of the Ni-Cu alloy [65]. The activity of d-electrons is lessened due to the fully occupied d-orbitals of Cu and unoccupied-d orbitals of Ni, augmenting the catalytic activity of the Ni-Cu alloy. The formation of coke is decreased by the addition of Cu to Ni catalysts.

### 2.5. Phenazine

**Scheme 4:**
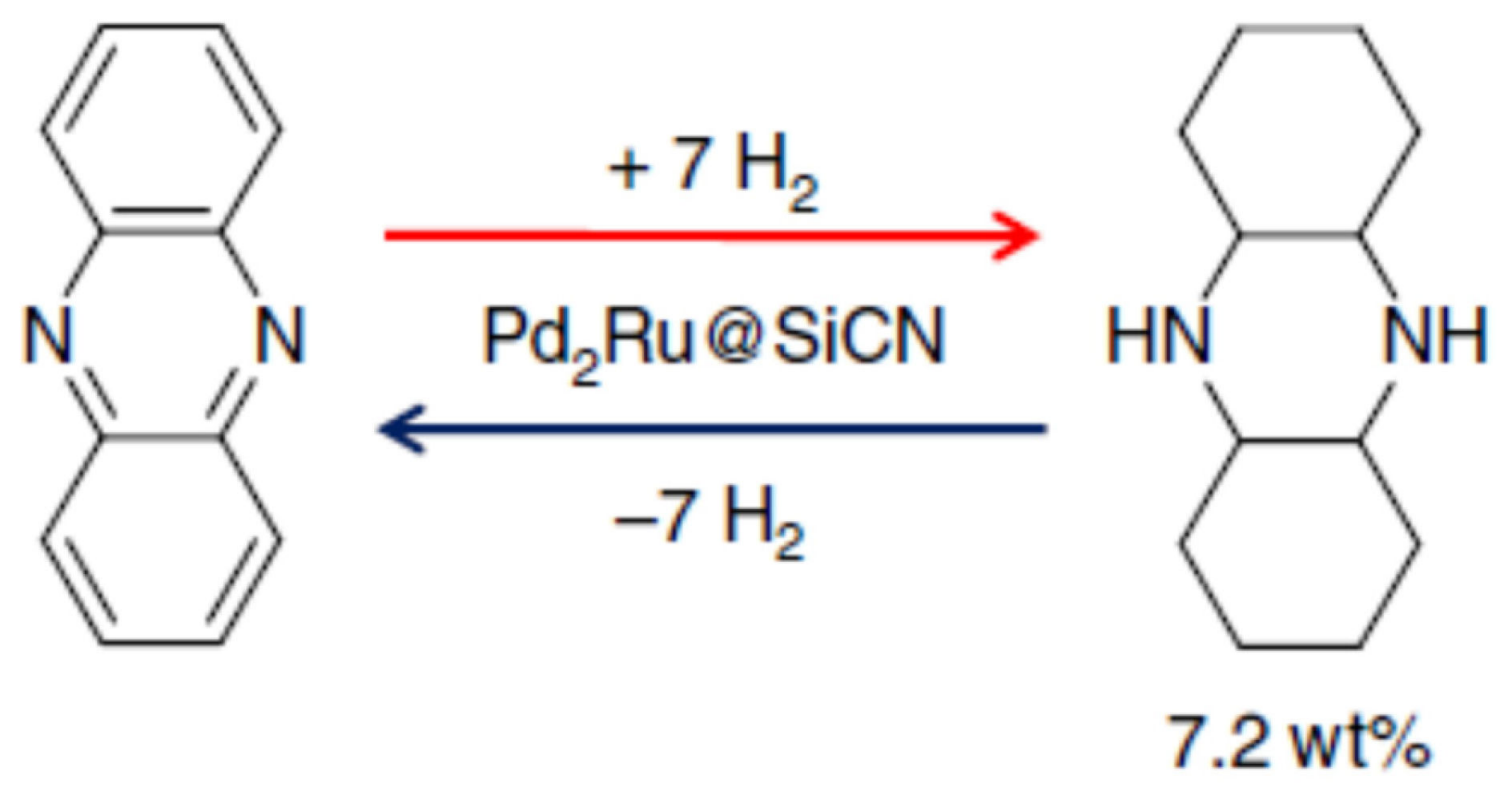
Hydrogenation and dehydrogenation of phenazine. Reproduced with permission from [66].

Lignin is a 3-dimensional biopolymer that was chemically modified to synthesise phenazine and was examined as an LOHC [66]. The hydrogenation of the compound was carried out at 115 °C, while the dehydrogenation was conducted in the presence of the Pd_2_Ru@SiCN catalyst as shown in Scheme 4. The amount of hydrogen collected was 6.19 weight %.

### 2.6. Indole Derivatives

**Figure 5:**
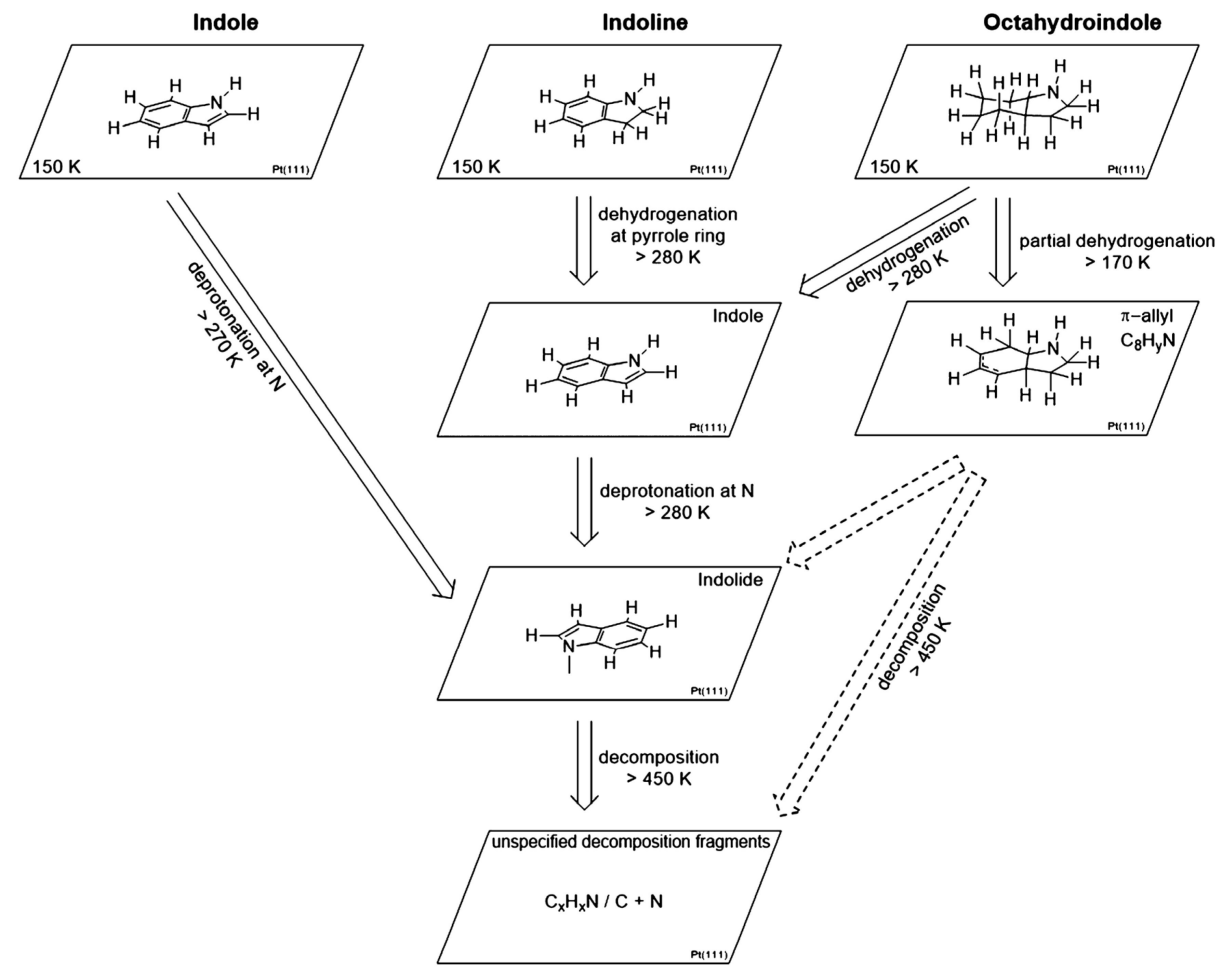
The reaction route for the LOHC on the Pt(111) surface. Reproduced with permission from [67].

Indole/octahydroindole possesses a capacity of hydrogen storage of 6.4%, while the storage capacity of indoline/indole is 1.7%. The dehydrogenation of indole, indoline, and octahydroindole on the Pt(111) surface was studied. The formation of a π-allyl species was observed in the dehydrogenation of octahydroindole without any formation of the indoline intermediate [67]. Again, the NH group was deprotonated above 300 K for all three molecules on the Pt(111) surface to form indolide. The molecule after the removal of the proton, however, loses the ability to desorb. The dehydrogenation of indoline and octahydroindole to indole occurred at 280 K. The reaction route is shown in Figure 5.

The α-indolinidylidene intermediate of indole interacts with the surface of the Pt(111) through the migration of hydrogen from C-2 to C-3 carbon [68]. The multilayers of deposited indole formed an ordered phase where indole molecules are bent towards the surface and undergo desorption at 221 K. The deprotonation of indole at the NH functional group takes place between 300 and 390 K to form a di-σ-indolide as an intermediate. A structural arrangement of a multilayer film formed on the Pt surface at 153 K due to the weak adsorption of indoline for H-bonding. The molecule also released a proton from the NH group along with the dehydrogenation of the five-membered ring above 300 K. These multilayers formed a low temperature by octahydroindole, which also showed the same phenomena above 300 °C.

N-ethyl indole (NEI) was reported as an LOHC molecule, in one study, which remains in a liquid state both as a carrier and also in its hydrogenated form [69]. One molecule of NEI can store four molecules of hydrogen. The hydrogenation was carried out at 190 °C in the presence of a Ru/Al_2_O_3_ catalyst. The hydrogenation proceeded as follows: NEI→2H−NEI→4H−NEI→8H−NEI. The formation of 6H NEI was hindered due to structural stress of the molecule. The molecules 2H-NEI and 4H-NEI were immediately hydrogenated after their formation. Kinetically stable intermediates failed to form like in the reaction with NEC, where 8H-NEC always formed as the kinetically stable intermediate [46]. The dehydrogenation was conducted with a 5 weight % of Pd/Al_2_O_3_ at 160–190 °C. Dehydrogenation also proceeded in the reverse order of hydrogenation. The hydrogen gas obtained was highly pure.

The compound 2-methyl indole (MID) was found to possess a hydrogen storage capacity of a 5.76 weight % and remained as a liquid at both the hydrogenated and dehydrogenated states. The dehydrogenation of 8H-methyl indole was carried out by a group with 5 wt% Pd/Al_2_O_3_, while the hydrogenation was conducted in the presence of 5 wt% Ru/Al_2_O_3_ catalysts [70]. The reaction rate was fastest at 170 °C. The reaction was completed at 130 °C within 130 min with the formation of two intermediates: 2H-methyl indole and 4H-methyl indole. The 6H-methyl indole species is kinetically unstable and was not detected. The 2H-methyl indole structure has double bonds in the five-membered ring, while the 4H-methyl indole structure has C-5, C-6, and C-7, C-8 double bonds hydrogenated in the benzene ring. The temperature of dehydrogenation was 160–200 °C, without the formation of any intermediates.

**Scheme 5:**
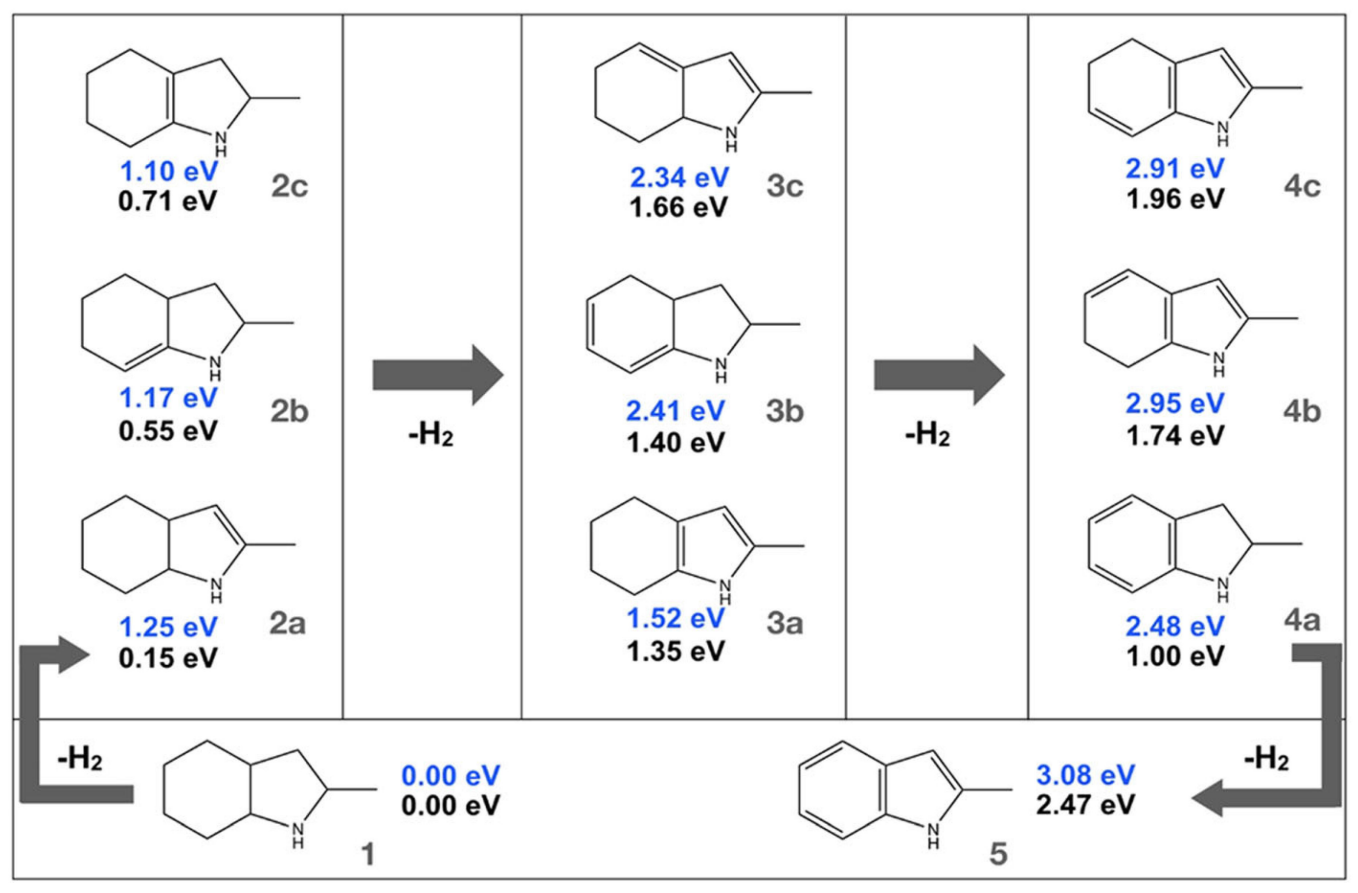
Dehydrogenation of the indole derivatives. Reproduced with permission from [71].

The dehydrogenation of substituted indoles (2-methyl indoline, 2-methyl indole, and 2-methyloctahydro indole) as LOHCs was studied on the Pt(III) surface as shown in Scheme 5 [71]. The dehydrogenation of the 2-substituted indole system revealed decomposition of the molecules during the process, indicating a low thermal stability as compared to their unsubstituted counterparts. Additionally, the dehydrogenation was completed for substituted indoles. The compound 2-methyl indoline was formed as an intermediate during the dehydrogenation of 2-methyl octahydroindole, establishing the fact that dehydrogenation is initiated at the six-membered ring.

The methyl group stabilizes the 2-methyl indoline based on electronic and steric effects. Two π-allylic intermediates are formed during the dehydrogenation of 2-methyloctahydro indole. In the gaseous phase, the removal of one mole of hydrogen forms the most stable isomer with the double bond placed between two rings of a molecule, and the isomers where the lone pair of electrons is in conjugation with the double bond. The removal of the next mole of hydrogenation formed the structure in which the five-membered ring was aromatised to a pyrrole unit, followed by the formation of a dehydrogenated six-membered ring after the final dehydrogenation. However, the stability of the products altered due to surface stabilization. The structure in which the conjugated π-system is adequately in contact with the atoms on the surface is the most stable. Again, a stable π-allylic intermediate is formed on the surface in the intermediate step between the double and single dehydrogenation step.

### 2.7. Pyridine Derivative

Piperidines have been used as LOHCs due to their low melting points, cheapness, and high capacity for hydrogen storage [72]. The dehydrogenation and hydrogenation of pyridine and 2-methyl pyridine was carried out using Pd and activated carbon as a catalyst [73] in one study. The acceptor-less dehydrogenation of 2-methyl piperidine to 2-picoline was conducted by using Pd/C (4 wt%), while the dehydrogenation of the same compound under solvent-free conditions was carried out in the presence of Pd(OAc)_2_/C. The system demonstrated a hydrogen storage capacity of 6.1 weight %. The 2,6-lutidine/2,6-dimethylpiperidine system has a hydrogen storage capacity of 5.3 weight %, which was also dehydrogenated effectively using the same catalyst system. Both the LOHC systems were found to be stable.

The dehydrogenation of perhydro 2-(n-methylbenzyl) pyridine was studied using a mesoporous catalyst Pd on Al_2_O_3_ prepared by the solvent deficient precipitation method [47,74]. The nanoparticles of Pd were composed of the Pd(111) plane which released hydrogen at a low rate in the primary stage, since the rate of diffusion was limited due to the pellet structure of the catalyst. However, the rate of evolution of hydrogen was enhanced later due to the good dispersion of Pd particles and the high surface area of the catalyst. The close packing of the Pd atoms of Pd nanoparticles on alumina in the thermodynamically stable Pd(111) facet aids in the adsorption of the heterocyclic compound. The adsorbed geometry is such that Pd molecules and hydrogen atoms are in close vicinity, which accelerates the rate of dehydrogenation as compared to Pd(110) and Pd(100) [75]. The movement of Pd particles is restricted by the alumina particles, thereby hindering the aggregation of Pd particles and thus retaining the dehydrogenation activity intact for four cycles.

### 2.8. Pyrrolidine

Butyl pyrrolidine is a promising candidate as an LOHC, as it is liquid in both hydrogenated and dehydrogenated states. It possesses a hydrogen weight % of 3.14 [76]. The catalytic dehydrogenation of butyl pyrrolidine was studied using an iridium pincer catalyst. The rate of dehydrogenation is comparatively faster than NEC due to the presence of a flexible n-butyl chain. Thus, this study revealed that the interaction of the metal centre of a catalyst with an LOHC is more significant than that of the C-H bond strength for an effective reaction.

## 3. Reactors Used for Dehydrogenation

**Figure 6:**
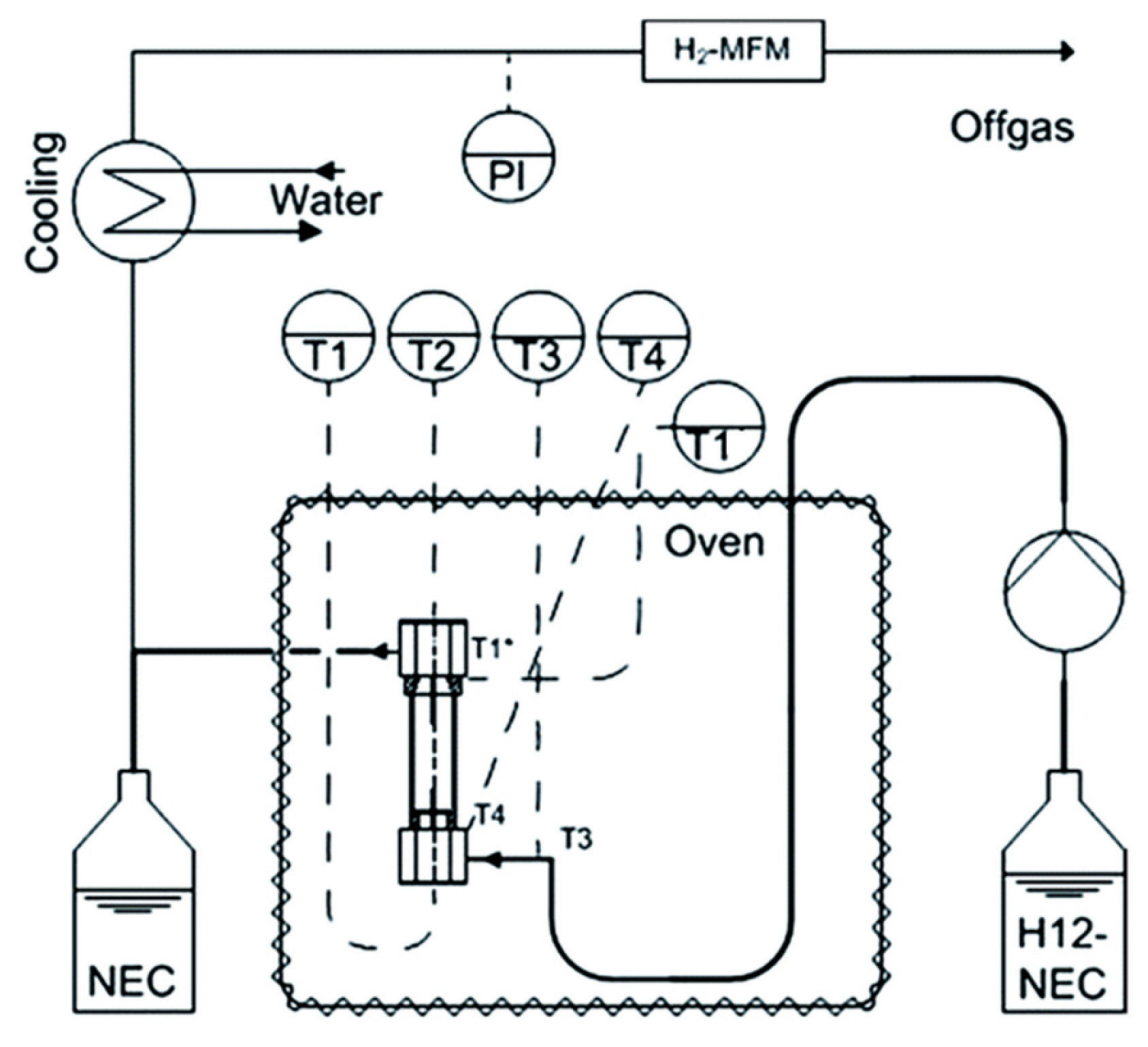
Schematic representation of the reactor. Reproduced with permission from [77].

The dehydrogenation process of hydrogenated LOHCs is complex, as the reaction heat should be effectively transported to the activation site of the catalysts. Additionally, a significant amount of hydrogen gas is evolved within the reactor. A reactor was fabricated, using selective electron beam melting (SEBM) instead of laser beams, in which a metal powder in the bed was melted locally using an electron beam for dehydrogenating LOHCs [77]. The reactor was layered with the simultaneous fabrication of the inner 3D structure and its walls. The electron beam was based on electromagnetic fields and a high speed was obtained, appropriate for Ti-6Al-4V powders. The set-up of the reactor is shown in Figure 6.

The rate of the reaction was high at an elevated temperature which is attributed to a high rate constant of the reaction under high-temperature conditions, and a faster propelling force to carry out the endothermic dehydrogenation reversible reaction. The initial dehydrogenation from H12-NEC to H8-NEC is the most feasible step, while further dehydrogenation steps are slothful. An enhancement in the rate of flow of LOHC decreases the generation of hydrogen as the residence time declines. The stability of the system is also very high.

## 4. Catalysts Used for Dehydrogenation

**Scheme 6:**
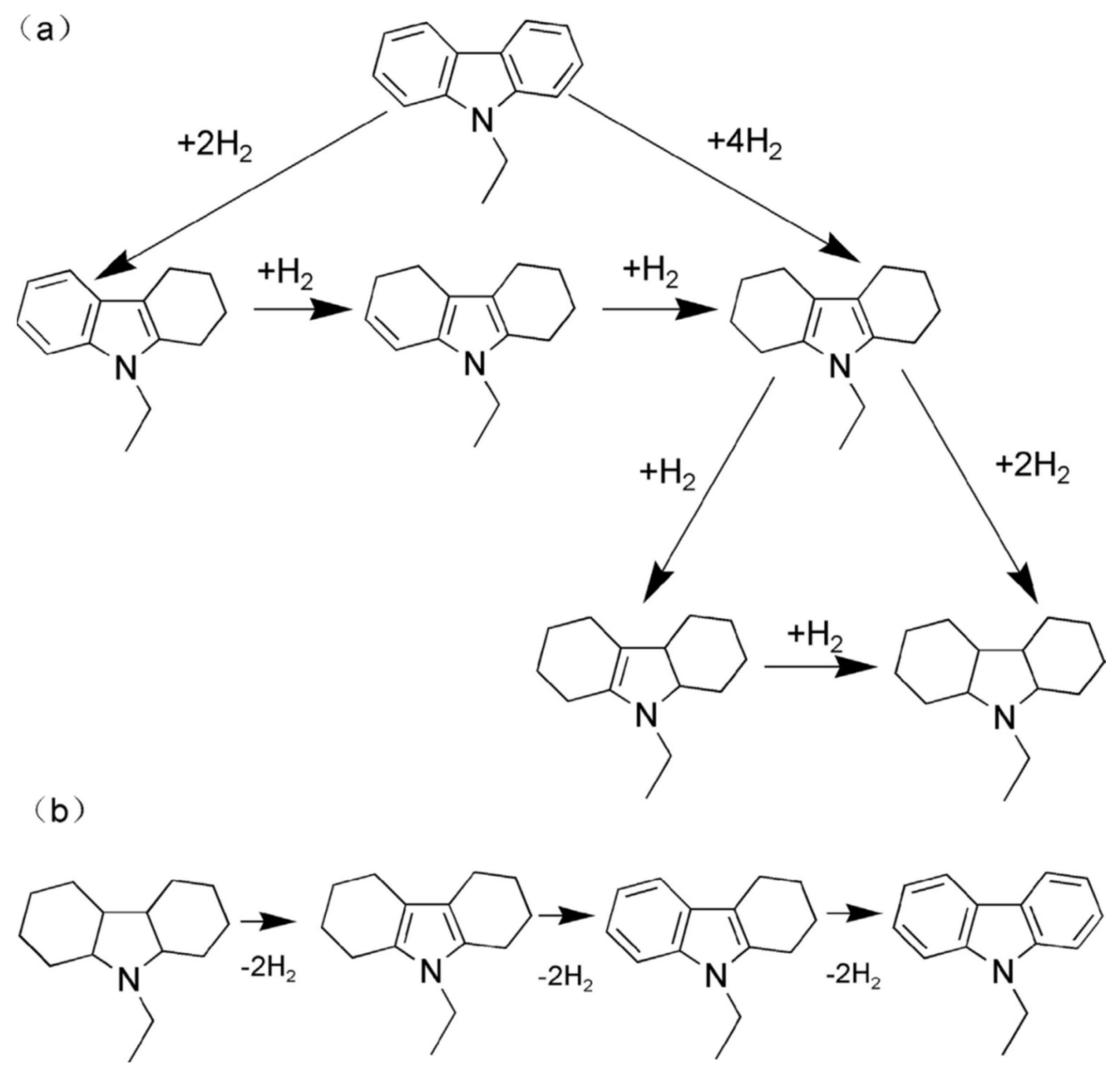
The process of (**a**) hydrogenation of NEC; (**b**) dehydrogenation of 12H-NEC. Reproduced with permission from [79].

The dehydrogenation of 12H-NEC is not feasible at atmospheric pressure, which has led to the development of different catalysts. Homogeneous catalysts, prepared from PCP pincer iridium complexes, failed to produce complete dehydrogenated products even after a prolonged reaction time [78]. A comparative study revealed that Pd supported on alumina was found to be effective as compared to Pt, Rh, and Ru [17]. The hydrogenation and dehydrogenation process are shown in Scheme 6.

It was found from a density-functional theory (DFT) analysis that the catalytic activity of Pd(111) for adsorption and dissociation is better as compared to Pd (100) and Pd(110) due to greater facet proportion [80]. The dehydrogenation process followed three stages as shown in Equations (3)–(5) [81]. The initial step is the removal of 2 moles of hydrogen from the five-membered ring of 12H-NEC to form 8H-NEC, followed by the removal of another 2 moles from one of the six-membered ring producing 4H-NEC which is a stable intermediate. The last stage is the elimination of hydrogen sluggishly to form NEC. The last stage is considered as the rate-limiting step [17].

(3) $$12H-NEC\to 8H-NEC+2H_{2}$$

(4) $$8H-NEC\to 4H-NEC+2H_{2}$$

(5) $$4H-NEC\to NEC+2H_{2}$$

In one report, hydrogen (600 mL) was obtained through the complete dehydrogenation of liquid H12-NEC (1 mL) and NEC (0.85 mL) upon reaching the ambient temperature. The high gas flow rate influences the mass transfer technique in catalyst pores. Continuous reactors are preferred to study and estimate the kinetics of a reaction, after reaching a steady-state condition, after a constant time-period of reaction. A study of the kinetics was conducted by substituting powder catalysts with catalyst pellets to prevent the fluctuations, the formation of bubbles, and pulsation associated with powder catalysts. The rate of diffusion depends on the particle size of catalysts which can be made constant by applying catalyst coating layers with different thicknesses. Coating methods include slurry coating, sol-gel coating, and a hybrid combination of both techniques. Different coating methods are: brush coating and spin coating for flat substrates; drop coating and dip coating for microreactors; as well as spray and electrostatic sol spray coating on various structures. In another study, the dehydrogenation of LOHCs was studied with Pd in combination with different metals (Ru, Pt, Cr, W, and Ge) supported on TiO_2_, of which W, Cr, and Ge show no activity [82]. Pd-Ru/TiO_2_ and Pd-Pt/TiO_2_ were effective at 195 °C after 6 h. The dehydrogenation was also carried out under microwave at 10 W with complete conversion in 100 min. Intermediates formed under microwave were 10H-NEC, 6H-NEC, and 2H-NEC. Additionally, the formation of side products like methane and ethane was suppressed. Microwaves are absorbed by the metal centres instead of the matrix composed of TiO_2_. A polarized monoatomic layer is created by the dissociation of hydrogen at the surface of metal nanoparticles. Thus, the selectivity of this hydrogenation process is improved because the metal centres are solely activated. The dehydrogenation of 12H-NEC was studied by using a 0.5 weight % of Pd on mesoporous MoO_3_, where the dispersed Pd particles offer active sites for the dissociation of H-H bonds and the movement of protons on the mesoporous surface [83]. The specific surface area of MoO_3_ is small for the absorption of hydrogen. The reaction rate was slow for the conversion of 4H-NEC to 8H-NEC. The conversion was low over mesoporous MoO_3_ due to a slow rate of dissociation of the H-H bond. The addition of Pd to mesoporous MoO_3_ enhanced the process of hydrogenation. In the same line, the dehydrogenation process of 12H-NEC was studied in the presence of eggshell (Pt/γ-alumina layer on α-alumina core) catalysts [17,84]. The coatings presented a homogeneous texture with cracks, although an 80 wt% of water was present in the dispersion. The thickness of the active catalyst layer was 33 mm and the dehydrogenation process was obtained at 260 °C. The diffusion rate of the substrate within the pores should be high to come into contact with the catalyst. The limitation for mass transfer is augmented at an elevated reaction temperature. The transition temperature shifts from kinetics to mass transfer when the coating is thinner, establishing a high dependency of the rate of reaction on the diffusion rate through the pores. The effect of pore diffusion can be prevented by considering two factors. One factor is by reducing the temperature and designing a thick coating of the catalyst. In contrast, the evolution of hydrogen will be decreased as the rate of dehydrogenation depends on the temperature. The next factor is to decrease the thickness of the catalyst layer. However, this diminishes the volumetric productivity of reactors, since the number of active catalysts per reactor volume decreases under this condition. Thus, for optimal efficacy of the dehydrogenation process, proper compatibility between the reaction temperature and the thickness of the layer should be maintained. In another study, the dehydrogenation of 12H-NEC was examined by supporting Pd on reduced graphene oxide, which exhibited a good performance by releasing a 5.79 weight % of hydrogen [85]. The catalysts exhibited good efficacy after five cycles. The nanoparticles of Pd are well dispersed on the two-dimensional plane with more active sites. Additionally, the contact between the substrate and the active site is more encouraging due to the open plane two-dimensional geometry. The interaction between Pd and hydrogen is weaker, thereby enhancing the desorption process [86]. However, the presence of oxidized Pd decreases the catalytic activity.

**Figure 7:**
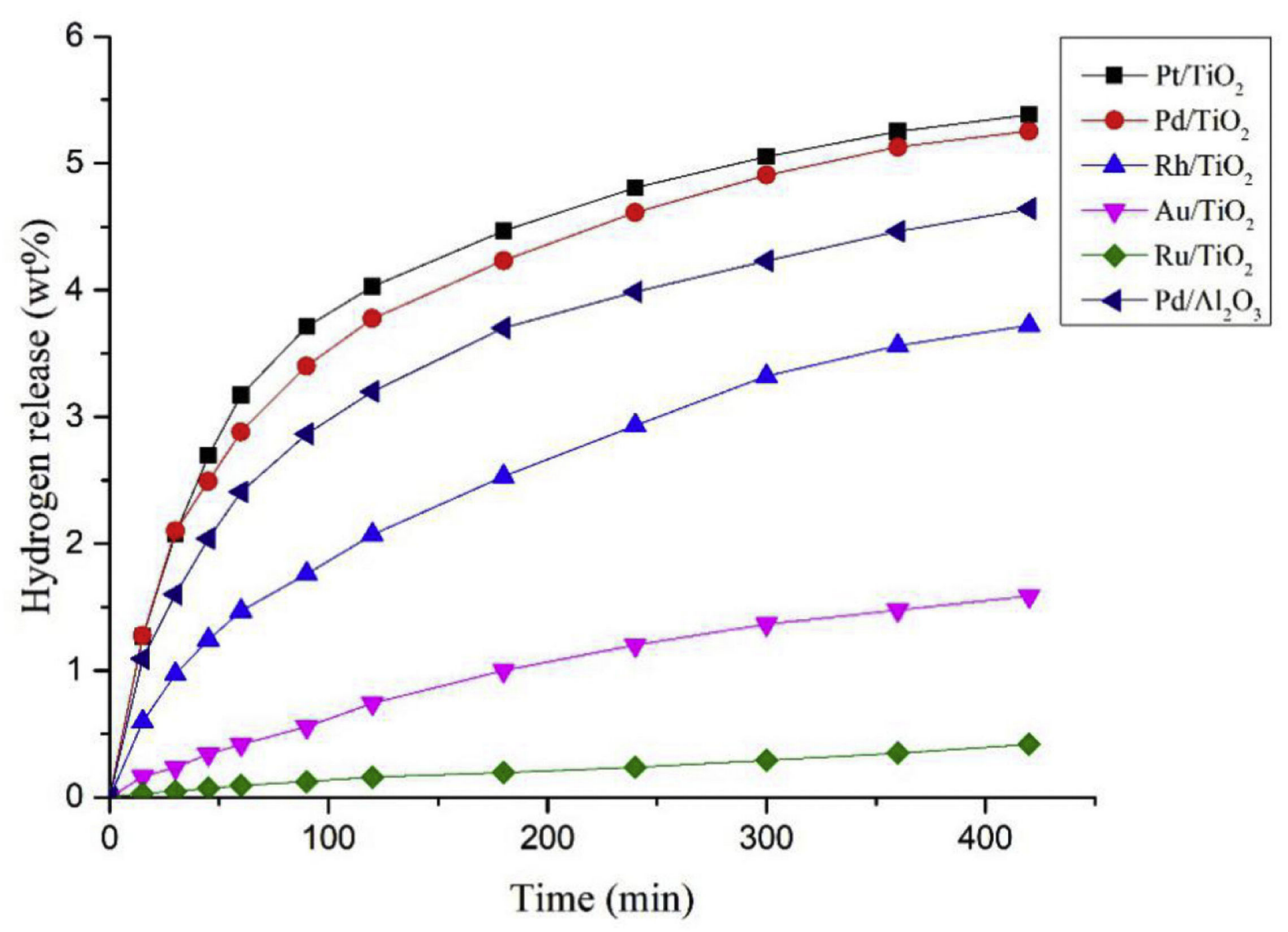
Amount of hydrogen released from the different catalyst. Reproduced with permission from [91].

The kinetics of a dehydrogenation process of 12H-NEC was studied by using noble metals supported on reduced graphene oxide surfaces [87]. Dominant facets of Pd(111), Pt(111), and Au (111) indicated a crystal orientation order. The binding energy of active metal and hydrogen atoms is decreased, facilitating the desorption of hydrogen gas after the process of dehydrogenation [17,88]. Pd particles (1.5–5 nm) were well dispersed on the reduced graphene oxide surface, enhancing the catalytic activity. The particle size of Pt was 2.24 nm, while that of Ru was 4.41 nm with crystals of irregular shapes which decreased their catalytic activity. Additionally, the agglomeration of Rh occurred as compared to the other examined catalysts. The reactivity of Au/reduced graphene oxide is lesser, while Ru/reduced graphene oxide and Rh/reduced graphene oxide released a lesser amount of hydrogen. Although the Pt/rGO was found to be an apposite dehydrogenating agent, it was less effective than Pd/rGO which liberated 5.79% of hydrogen at 453 K in 4.5 h of reaction time. The activity of the catalyst was dictated by the temperature at which the reaction was conducted, as the rate and degree of the reaction were augmented with an increase in temperature. The compound 8H-NEC was formed faster with the slow progress of dehydrogenation of 8H-NEC. Palladium nanoparticles were supported on carbon nanotubes and used as heterogeneous catalysts for the dehydrogenation of 12H-NEC in one study [89]. Pd nanoparticles were distributed homogeneously on carbon nanotubes surface, thus increasing the efficacy of the catalyst. The liberation of hydrogen increased with an increase in temperature and was at a maximum at 533 K with a 3 wt % of Pd on carbon nanotubes. The catalyst was reused which generated a 5.6 wt % of hydrogen in the fifth cycle. In one report, the Pt/Al_2_O_3_ catalyst was improved by adding one noble metal (Pd) and one non-noble metal (Cr) for the dehydrogenation of 12H-NEC [90]. Complete dehydrogenation was not possible with the Pt/Al_2_O_3_ catalyst, while the reaction was 100% complete with the Pt-Cr/Al_2_O_3_ and Pt-Pd/Al_2_O_3_ catalyst. The selectivity of these bimetallic catalysts was improved with the formation of a low concentration of ethane and methane as by-products during the evolution of hydrogen. The presence of by-products like methane and ethane decreases the capability of hydrogen storage; hence, the formation of these gases should be averted. A comparative study between the two catalysts showed that Pt-Cr/Al_2_O_3_ was preferable, as it was associated with the low liberation of the by-products and belonged to the non-noble class, with a similar potential to the noble gas metals. These Pt nanoparticles were stabilized by the incorporation of a Cr atom which in turn enhanced the power of the catalyst. In another report, M/TiO_2_ was used as a catalyst, where M = Pd, Rh, Ru, Pt, and Au, for the dehydrogenation of 12H-NEC, among which Pt/TiO_2_ exhibited the highest selectivity and a faster rate of dehydrogenation [91]. The results revealed that Pt in combination with TiO_2_ was a better catalyst than Pd as shown in Figure 7. However, Pd has been established as a superior dehydrogenation catalyst [92]. Pt exhibited a better performance because TiO_2_ belongs to a class of special semiconductors that can deliver additional electronic charges to Pt. The electron-rich Pt conducted the dehydrogenation process in a facile manner. It was found that although the active sites on Pt were lesser as compared to Pd, it was more active. A comparative study between Pd/TiO_2_ and Pd/Al_2_O_3_ indicated that the former has a higher catalytic activity due to the transfer of electron charge to Pd clusters from TiO_2_. The intermediate strength of binding between the molecules and the active centres on the surface of catalysts dictates the activity of the catalysts. A weak strength indicated that the molecules were less activated and the reaction was slower. In contrast, a strong intermediate strength indicated that the products failed to be desorbed from the catalytic active sites, thus blocking the requisite sites for the reaction. This intermediate strength was found to be optimal for Pt and Pd.

A dehydrogenation catalyst was synthesized by the homogeneous dispersion of nanoparticles of Pd-Cu on the surface of reduced graphene oxide, where Pd_1.2_Cu/reduced graphene oxide was found to be effective for complete conversion to NEC within a reaction time of 7 h at 453 K [93,94,95]. The observed reaction rate was similar to that of Pd/reduced graphene oxide. However, as the amount of Pd was reduced in this study, the cost of the catalyst was substantially decreased. The activity of the catalysts was higher with a smaller size. An enhancement in the amount of Cu to 50% does not affect the binding energy of Pd-3d. However, a higher amount of Cu (more than 50%) initiates the transfer of electrons from Pd to Cu, resulting in a decreased dehydrogenation rate. The electron distribution in the outer shell of Pd is also affected by the ratio of the Pd and Cu.

## 5. Application

**Figure 8:**
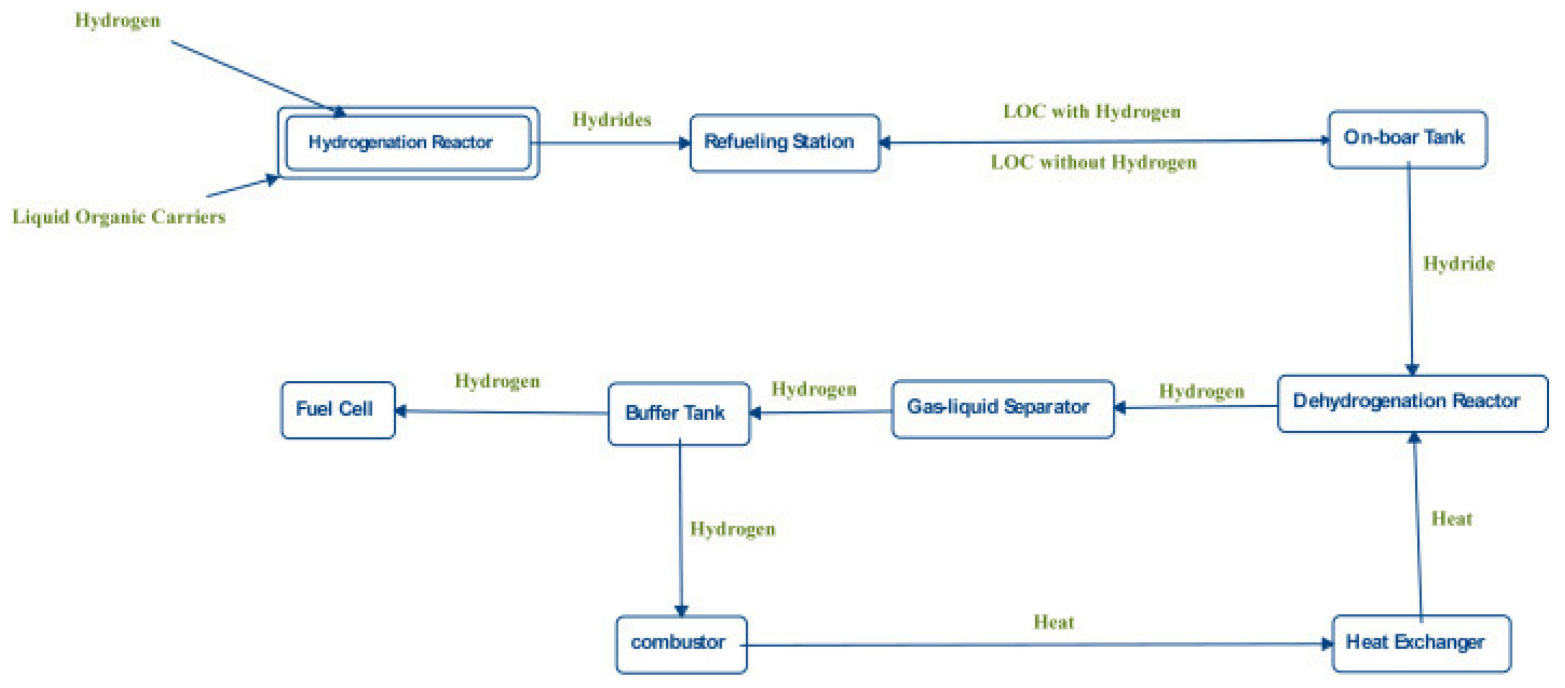
Flow-chart of the application of the LOHC. Reproduced with permission from [31].

The hydrogenated LOHC can be transported by vehicles and delivered to refuelling stations. The hydrogenated LOHC is stored in tanks and on a requirement can be transported to reactors. A reactor is heated from the heat generated by fuel cells during the dehydrogenation of the samples. Since a mixture of gas and liquid is obtained, a separator of gas and liquid is placed after the reactor. Gaseous hydrogen moving out of the separator is stored in a buffer tank for acquiring the pressure desirable to operate the fuel cells. A portion of hydrogen moves to the fuel cells, while the other part moves to combustors for generating heat for the reactors. The heat of combustion is used to heat the water produced from the fuel cells. Some of the heat can be employed in the reactors with the assistance of a heat exchanger. The regenerated carrier is directed into separate containers for further hydrogenation, as illustrated in Figure 8.

**Figure 9:**
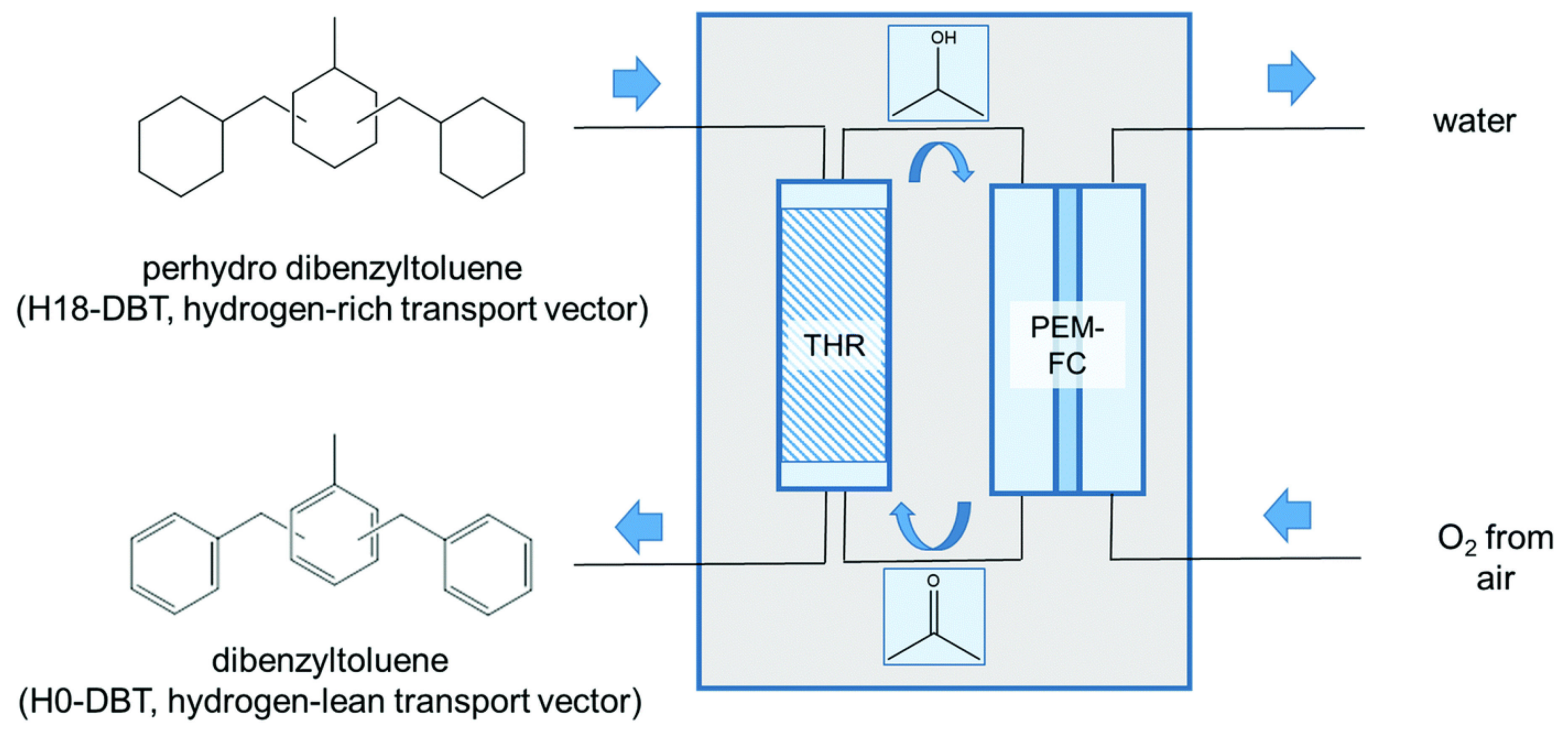
Generation of electricity by the transfer hydrogenation process. Reproduced with permission from [10].

A polymer electrolyte membrane fuel cell (PEMFC) (using 2-propanol/acetone mixture) was operated by the transfer hydrogenation of H18-DBT using acetone [10]. Thus, hydrogen obtained by the dehydrogenation of H18-DBT was activated and used for the formation of 2-propanol from acetone at 190 °C. The rate of dehydrogenation was mild, since the liberated hydrogen was continuously used for the hydrogenation of acetone. The system of transfer hydrogenation should operate in a counter-current mode. The acetone vapour was allowed to diffuse through the catalyst surface at the bottom of the reactor where the LOHC carrier flows as a counter current. Hydrogen was transferred to acetone at the bottom from LOHC. The 2-propanol vapour at the top of the reactor mixes with H18-DBT for the conversion of any trace acetone which might be present as shown in Figure 9. Thus, 59% of the electricity was generated from hydrogen bound to the LOHC at a temperature <200 °C. The transfer of hydrogen to acetone from H18-DBT was a thermoneutral process. Thus, external heating could be avoided. A stack of 20 direct methanol fuel cells was investigated using 2-propanol, which generated an open circuit potential (OCP) of 15.56 V with a power density of 25.3 mW cm^−2^ [10,96,97,98]. The OCP per unit cell was 802 mV. The exothermic formation of water was balanced by the endothermic release of protons. The catalyst used for transfer hydrogenation was Pt/C. This system avoided the formation of molecular hydrogen during the complete process, indicating the safety feature.

## 6. Technoeconomic Analysis

### 6.1. Technical Analysis

The hydrogen transportation and storage efficiency is described using Equation (6) [99,100,101,102].

(6) $$\eta_{H_{2}}=\frac{E_{hyd}+E_{H_{2,use}}}{E_{H_{2,in}}+E_{storage}+E_{dhyd}}$$

where

$\eta_{H_{2}}$ = hydrogen transportation and storage efficiency;
$E_{H_{2,use}}$ = useable hydrogen output energy content;
$E_{H_{2,in}}$ = useable hydrogen input energy content;
$E_{storage}$ = energy demand of the storage system;
$E_{hyd}$ = energy of hydrogenation;
$E_{dhyd}$ = energy of dehydrogenation.

The hydrogen transportation and storage efficiency indicate the ratio of the energy content of the useable hydrogen output to that of the hydrogen input. The efficiency depends on the energy demand of the storage system and the high heating value of hydrogen. An extra output is obtained, as hydrogenation is an exothermic process. This additional output can be used to enhance the efficiency of the system.

The chain efficiency and transport efficiency are expressed as Equations (7) and (8), respectively [102].

(7) $$\eta_{chain}=\frac{E_{fuel\;cell}}{E_{electrolysis}+E_{storage}+E_{transport}}$$

(8) $$\eta_{transport}=\frac{E_{H_{2,use}}-E_{transport}}{E_{H_{2,use}}}$$

where

$\eta_{chain}$ = chain efficiency;
$\eta_{transport}$ = transport efficiency;
$E_{transport}$ = energy consumption during transport;
$E_{electrolysis}$ = energy consumption during electrolysis;
$E_{storage}$ = energy consumption during storage;
$E_{fuel\;cell}$ = energy output of the fuel cell.

### 6.2. Economic Analysis

An increase in capacity escalates the investment cost of a conversion plant. A high throughput reduces the specific costs at the same time. The cost of design and the fabrication of hydrogenation and dehydrogenation reactors are given by Equations (9)–(11) [102,103,104]. These equations were used for dibenzyltoluene (DBT) via fitting the curves of different sizes at varied costs.

(9) $$C_{des}=C_{base}{\left(\frac{S_{des}}{S_{base}}\right)}^{SF}$$

(10) $$C_{hyd}={2840.81\;P}_{hyd.max}^{-0.375}\frac{{STY}_{DBT}}{{STY}_{LOHC}}$$

(11) $$C_{dhyd}={3122.83\;P}_{hyd.max}^{-0.375}\frac{{STY}_{DBT}}{{STY}_{LOHC}}$$

where,

$C_{des}$ = the cost of design;
$C_{hyd}$ = the cost of hydrogenation reactors;
$C_{dhyd}$ = the cost of dehydrogenation reactors;
STY = space, time, and yield;
$P_{hyd.max}$ = maximum power rating in kW;
SF = the scaling factor.

The chemical engineering plant cost index is used as a basis to calculate the adjustment of costs of the equipment installed [104]. Products obtained per time and volume are denoted by the space–time–yield parameter. The size of the reactors is compared using this parameter for different LOHCs using Equation (12). Technical operations require the equilibrium conversion of dehydrogenation for addressing local decoupling. The loaded LOHC moves back to the hydrogenation site when the conversion is <100%. This reduces the useable storage density.

(12) $$STY=\frac{n_{A}\chi_{A}M_{A}}{V_{Ao}t_{R}}$$

where

$n_{A}$ = moles of the target (A) per mole of the reactant (A_o_);
$\chi_{A}$ = the equilibrium conversion;
$M_{A}$ = the molar mass;
$V_{Ao}$ = the volume of 1 mole of reactants;
$t_{R}$ = reaction time.

The fixed capital investment is determined by multiplying the design cost of the equipment by a ratio factor. This accounts for additional costs such as legal and installation expenditures. Additional costs of working capital and contingencies are accounted for as the total costs.

The length of transport of the produced electricity to the area of consumption is another area of investment. The LOHC process chain includes the following components: (i) the production of hydrogen from green electricity, (ii) hydrogenation, (iii) storage and transportation of LOHCs, (iv) dehydrogenation, and (v) the operation of fuel cells or the generation of electricity. Hydrogenation requires high pressure. Thus, it can be replaced by electrolysis under high pressure to decrease the cost of compression. The product stream remains saturated with hydrogen after hydrogenation. Hence, hydrogen released during decompression should be stored to reduce the cost. The LOHC that did not undergo dehydrogenation should be recirculated with the LOHC in the carrier. Thus, the storage capacity for transport is diminished.

**Figure 10:**
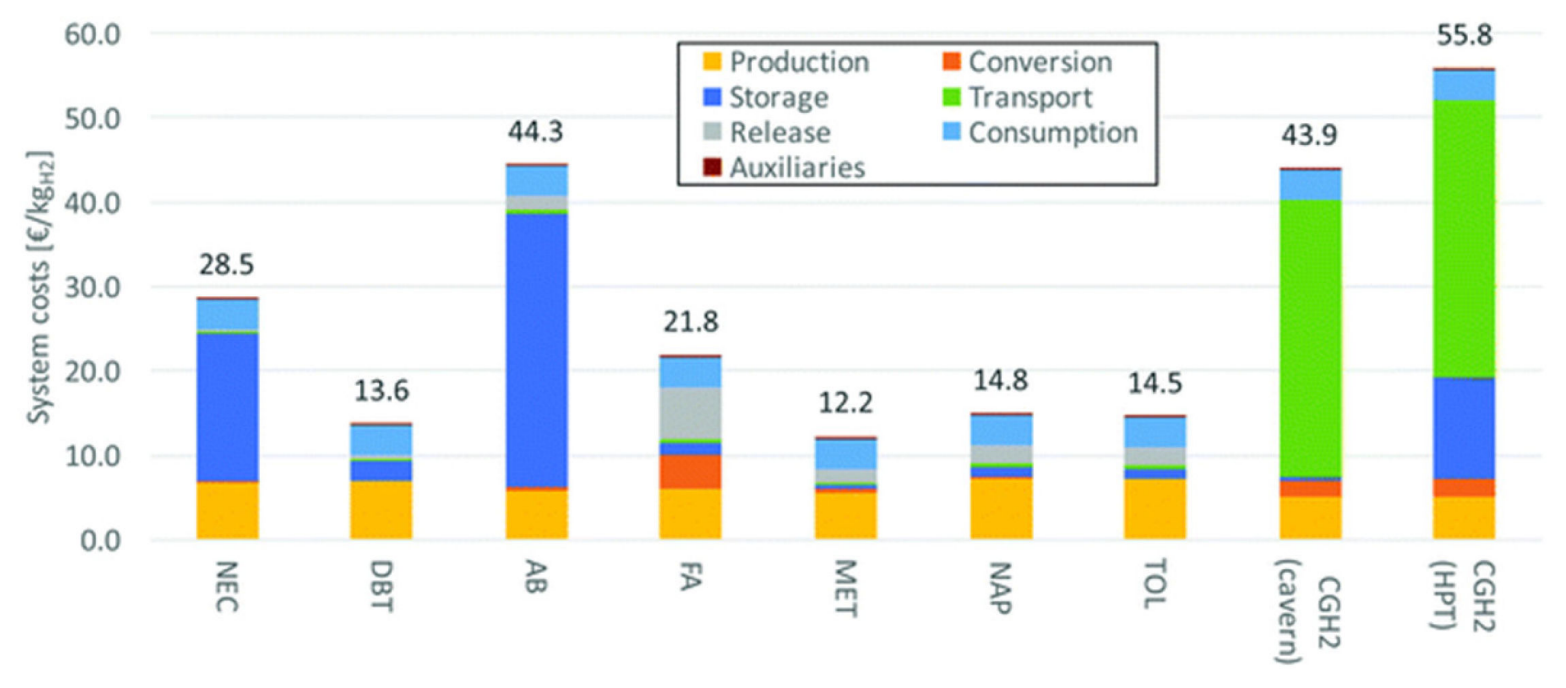
System costs covering dehydrogenation heating (NEC: N-ethylcarbazole, DBT: dibenzyltoluene, AB: 1,2-dihydro-1,2-azaborine, FA: formic acid, MET: methanol, NAP: naphthalene, TOL: toluene, CGH2: compressed hydrogen gas, HPT: high-pressure tank). Reproduced with permission from [102].

**Figure 11:**
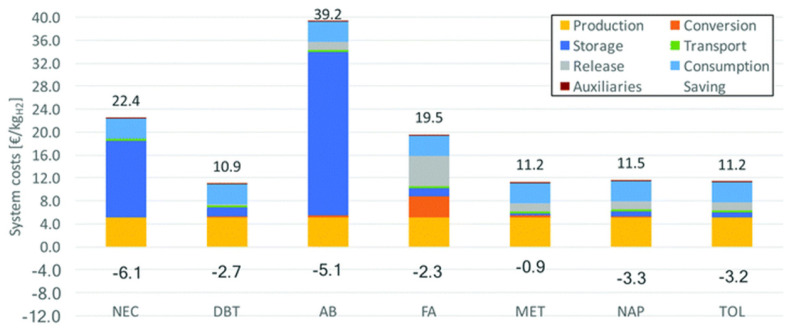
System costs covering dehydrogenation heating using the waste heat of fuel cells (NEC: N-ethylcarbazole, DBT: dibenzyltoluene, AB: 1,2-dihydro-1,2-azaborine, FA: formic acid, MET: methanol, NAP: naphthalene, TOL: toluene). Reproduced with permission from [102].

Methanol and 1,2-dihydro-1,2-azaborine are suitable LOHCs as they require a low heat for dehydrogenation. However, the purification of hydrogen is required for these two compounds. DBT and NEC are acceptable LOHCs, while formic acid is expensive as it requires a high-energy distillation step. The waste heat from fuel cells can be used for the dehydrogenation of LOHCs. Thus, DBT and NEC are suitable candidates as they do not require further purification and have a high compressed hydrogen efficiency. Methanol is an economical LOHC, since it is a cheap raw material. The transport of compressed hydrogen is costly through pipelines but is economical through ships. Using fuel cells for acquiring the heat required for dehydrogenation enhances the efficiency and reduces the budget. The economic analysis of different LOHCs is shown in Figure 10 and Figure 11.

The electrolysis of alkaline water produces on-site hydrogen with 62% efficiency calculated based on the lower heating value of hydrogen [105]. The total costs of investment for 10 and 2.5 MW hydrogen were EUR 15 and 5 million, respectively. Investment costs include grid connection, building, installation, and piping. The operation and maintenance costs were 5% of investment costs, while the durability of an electrolyser is 15 years. The low-temperature heat and oxygen obtained from water electrolysis did not have additional costs.

Although the delivery cost of LOHCs is higher, LOHCs have other advantages. A significant amount of hydrogen can be stored at a considerable cost. Moreover, the hydrogen obtained from certain LOHCs is pure and requires no further purification step. However, this is not applicable for all LOHCs, as hydrogen produced from some LOHCs requires further purification. Thus, the cost of the purification steps should also be considered. The key concept is heat integration. The site of the hydrogen source should use the heat obtained from hydrogenation, while the hydrogen-consuming site should use cheap source of external heat for dehydrogenation. These steps would reduce the cost of the LOHC supply. However, the use of direct electrical heating is economical with the availability of cheap electricity.

## 7. Conclusions and Future Perspectives

The ever-increasing demand for energy has compelled humanity to seek alternative energy sources. Moreover, present non-renewable energy sources are becoming depleted with time. The global warming issues have driven researchers to explore clean and green energy sources. Keeping this in mind, energy generated from stored hydrogen has become an attractive option as a clean energy source. However, the transportation and storage of hydrogen are expensive and risky. Thus, LOHCs are a key solution for the cheap and facile storage and transportation of hydrogen. These molecules can be easily dehydrogenated and used as energy sources. Research has been conducted to find suitable molecules for feasible and practical applications. This research has summarized the potential molecules as LOHC candidates, their performances, and their applications.

The LOHC molecules have been discussed based on their varied characteristics, such as stability, storage capability, catalysts used, and reusability. The technology associated with LOHCs demands huge development. The stability of LOHCs can be enhanced by lowering the enthalpy of dehydrogenation of these molecules. Hence, studies encompassing thermodynamic tuning of both the hydrogenation and dehydrogenation processes are required. The use of catalysts lowers the activation energy of dehydrogenation of LOHC molecules. This results in the requirement of a low dehydrogenation temperature. Future research should be directed toward investigating efficient catalysts for the dehydrogenation of LOHC molecules at low temperatures and make the system economically viable.

The asset of LOHCs is that they resemble crude oil; hence, the existing systems can be used for the generation of energy. Therefore, dibenzyl toluene and toluene as LOHC candidates exhibit high prospects for mass commercial production. The application of LOHC molecules as energy sources should be further explored. The reactors for dehydrogenation can be improved for making the reaction conditions more feasible.

The toxicity of LOHC molecules should be minimal to conform with environmental concerns. Since these LOHC molecules are well-studied, it is easier to handle the risks of these molecules as compared to crude oil. LOHC should always be computationally designed considering the eco-toxicological profiles like the mutagenic, carcinogenic, and toxic profiles. However, these parameters are challenging to predict by the quantitative structure–activity relationship (QSAR). There has been a dearth of computer modelling studies, including biodegradability, efficacy, and toxicity, for the search of apposite catalysts for the dehydrogenation of LOHC molecules. Additionally, life cycle analysis and economic analysis of the LOHC technology should be studied to make the system sustainable. LOHC systems require transportation, hydrogenation, and dehydrogenation.

## Figures and Tables

**Table 1:** Thermodynamic properties of LOHCs. Reproduced with permission from [13].

LOHC	Free Energy of Reaction	Enthalpy of Reaction
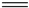	−100.4	−136.3
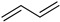	−83.3	−118.4
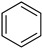	−32.6	−68.7
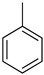	−31.6	−68.3
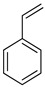	−43.6	−79.7
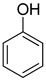	−25.6	−63.3
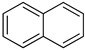	−20.5	−66.3
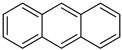	−19.6	−66.9
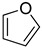	−40.3	−74.7
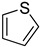	−40.4	−74.6
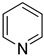	−29.5	−63.1
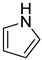	−22.8	−55.9
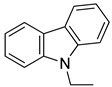	−18.2	−53.2

**Table 2:** Few examples of common LOHC molecules.

Unsaturated Compound	Saturated Compound	Gravimetric H_2_ Carrying Capacity (%)
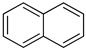 Naphthalene	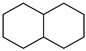 Decaline	7.5
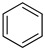 Benzene	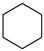 Cyclohexane	7.5
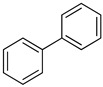 Biphenyl	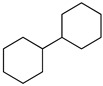 Bicyclohexyl	7.5
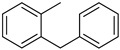 MLH	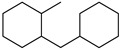 H_12_-MLH	7.0
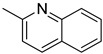 Quinaldine	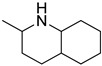 H_10_-Quinaldine	7.0
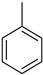 Toluene	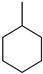 Methyl cyclohexane	6.5
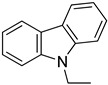 Ethyl carbazole	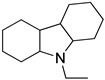 H_12_-ethyl carbazole	6.0
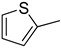 Methyl thiophene	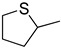 H_4_-methyl thiophene	5.5
